# Initiation of RNA Polymerization and Polymerase Encapsidation by a Small dsRNA Virus

**DOI:** 10.1371/journal.ppat.1005523

**Published:** 2016-04-14

**Authors:** Aaron M. Collier, Outi L. Lyytinen, Yusong R. Guo, Yukimatsu Toh, Minna M. Poranen, Yizhi J. Tao

**Affiliations:** 1 Department of BioSciences, Rice University, Houston, Texas, United States of America; 2 Department of Biosciences, University of Helsinki, Helsinki, Finland; University of Wisconsin - Madison, UNITED STATES

## Abstract

During the replication cycle of double-stranded (ds) RNA viruses, the viral RNA-dependent RNA polymerase (RdRP) replicates and transcribes the viral genome from within the viral capsid. How the RdRP molecules are packaged within the virion and how they function within the confines of an intact capsid are intriguing questions with answers that most likely vary across the different dsRNA virus families. In this study, we have determined a 2.4 Å resolution structure of an RdRP from the human picobirnavirus (hPBV). In addition to the conserved polymerase fold, the hPBV RdRP possesses a highly flexible 24 amino acid loop structure located near the C-terminus of the protein that is inserted into its active site. *In vitro* RNA polymerization assays and site-directed mutagenesis showed that: (1) the hPBV RdRP is fully active using both ssRNA and dsRNA templates; (2) the insertion loop likely functions as an assembly platform for the priming nucleotide to allow *de novo* initiation; (3) RNA transcription by the hPBV RdRP proceeds in a semi-conservative manner; and (4) the preference of virus-specific RNA during transcription is dictated by the lower melting temperature associated with the terminal sequences. Co-expression of the hPBV RdRP and the capsid protein (CP) indicated that, under the conditions used, the RdRP could not be incorporated into the recombinant capsids in the absence of the viral genome. Additionally, the hPBV RdRP exhibited higher affinity towards the conserved 5’-terminal sequence of the viral RNA, suggesting that the RdRP molecules may be encapsidated through their specific binding to the viral RNAs during assembly.

## Author Summary

Viral polymerases replicate the genome of the virus, which is essential for the synthesis of the progeny. All double-stranded (dsRNA) viruses have virion-associated polymerases that catalyze RNA synthesis within an intact capsid. Picobirnavirus (PBV) is a small dsRNA virus, and it has been shown that the capsid of PBV possesses an unusual architecture suggesting an uncommon assembly pathway that it could potentially share with another group of small dsRNA viruses called partitiviruses. We have performed both structural and functional studies to look at how the PBV polymerase performs its function and how these molecules are placed within the capsid. The PBV RdRP structure was solved and revealed that the RdRP has an interesting loop structure in its interior. Additionally, activity assays showed that the RdRP possesses catalytic activity in the absence of other viral proteins. Removing the loop structure was found to change the way that the RdRP initiates RNA replication. Further experiments also showed that the RdRP did not interact with the viral capsid protein (CP) but had a strong affinity for a conserved terminal sequence of the PBV genome. This suggests that in PBV the RdRP may be encapsidated based upon both the RdRP and CP co-interacting with the viral genome.

## Introduction

Double-stranded (ds) RNA viruses are a diverse group of viruses that vary widely in host range (humans, animals, plants, fungi, and bacteria), genome segment number (one to twelve), and in the number of capsid layers, with many of them considered important pathogens of either agriculture or human health. A common feature of dsRNA viruses is that their capsid associated polymerase performs both of its functions, namely replicating as well as transcribing the viral genome, from within the confines of the virus capsid. This sequestration of the polymerase and the dsRNA genome prevents the activation of the host’s RNA induced antiviral response [1].

During the viral replication cycle, dsRNA viruses have been shown to encapsidate up to twelve RNA-dependent RNA polymerase (RdRP) molecules in each virus particle [2–5] To date, several different mechanisms of incorporating the RdRP molecules into the capsid have been identified. Those that possess multi-layered capsids, such as the bacteriophage ϕ6, rotavirus, and reovirus, as well as the single-layered capsids of cypoviruses have been shown to attach their polymerase molecules to the inner surface of the capsid through direct protein-protein interactions [6–10], suggesting that non-covalent protein-protein interaction plays an important role in RdRP incorporation. A number of single-shelled dsRNA viruses, such as the yeast L-A virus, express their polymerase as a capsid protein (CP)-RdRP (*gag-pol*) fusion protein, which is then incorporated into viral particles as a minor structural component during capsid assembly [11,12]. With few exceptions, most notably the polymerase of bacteriophage ϕ6 [13], polymerases from dsRNA viruses are not fully active when their respective capsid proteins are not present [14–16]. It has been proposed that the dependence of polymerase activity on the presence of capsid proteins may help to ensure that dsRNA products are preferentially produced only within a capsid enclosure [17].

The crystal structures of the RdRPs from several dsRNA viruses (*i*.*e*. ϕ6, reovirus, rotavirus, and birnavirus) have been determined, and all have been found to contain a core polymerase domain with a right-hand shape [18–21]. In reovirus and rotavirus polymerases, which catalyze conservative RNA transcription, possess elaborate N- and C-terminal domains that interact with the core polymerase domain, thus creating a cage-like structure with four channels leading in and out of the active site at the center of the molecule [22]. These polymerases also possess an mRNA cap binding site that may facilitate the initiation of viral RNA transcription [19,21]. In contrast, the ϕ6 and birnavirus polymerases, which produce RNA transcripts in a semi-conservative manner, are relatively smaller in size with a structure containing only three active site channels. Distinct structural features have been identified in the RdRPs of ϕ6, reovirus and rotavirus that function as a structural platform for the binding of a single priming nucleotide to allow for *de novo* initiation of RNA synthesis [18,19,21,23,24].

Picobirnaviruses (PBV) are small, non-enveloped dsRNA viruses infecting a wide range of mammalian and avian species [25–29]. Human PBV (hPBV) has been identified on almost every continent, and has been associated with acute gastroenteritis primarily in children and people that are immunocompromised [30,31]. It has a bi-segmented genome with the genome segment two (PBV2) encoding the viral RdRP and the genome segment one (PBV1) encoding the CP and a protein of unknown function [32]. The crystal structure of a rabbit PBV virus-like particle (VLP) shows that the capsid possesses T = 1 icosahedral symmetry in which the asymmetric unit is a dimer. Such an organization is sometimes referred to as “T = 2” structure and is unique for dsRNA viruses [33]. However, the overall capsid organization of PBV appears to be somewhat different from the organization of the larger dsRNA viruses like reoviruses [34]. Instead of having side-by-side CP dimers clustering around the 5-fold symmetry axes (*i*.*e*. CP decamers), the PBV capsid is made of diamond-shaped dimers of dimers (*i*.*e*. CP tetramers). Such an unusual capsid organization has also been observed in fungal infecting partitiviruses, another family of dsRNA viruses with a bi-segmented genome [35,36]. The CPs for PBV and partitiviruses are small in size, with a structural fold that somewhat differs from those found in larger dsRNA viruses such as reoviruses and rotaviruses. Interestingly, the major capsid protein P1 of cystoviruses likely also forms tetramers, but the geometric shape of such tetramers and the structural fold of the P1 are somewhat different from those of PBV and partivirus CPs [37,38].

To elucidate the mechanisms of RNA replication, transcription, and RdRP encapsidation by this group of largely uncharacterized, small dsRNA viruses, we have determined the structure of the hPBV RdRP and systematically characterized its biochemical and enzymatic activities. The hPBV RdRP possesses a canonical polymerase fold with a 24 amino acids (aa) long C-terminal insertion loop structure that partially occupies the active site of the polymerase. A hPBV RdRP lacking this insertion loop, ΔLOOP, was subsequently generated to determine the functional role of this structure. Both the wild-type (WT) and ΔLOOP RdRPs are capable of RNA synthesis using both homologous and heterologous, single- and double-stranded RNA templates in the absence of the CP. However, while the WT RdRP utilizes a *de novo* initiation mechanism for RNA synthesis, the ΔLOOP could only initiate RNA replication through back-priming, suggesting that the insertion loop serves as a platform for initiation. For transcription the hPBV polymerase uses a semi-conservative mechanism in which the positive-strand of the template dsRNA is dislodged from the duplex on the RdRP surface as the negative-strands enters into the template tunnel and the newly produced transcript forms a duplex with the negative-sense RNA strand. We also demonstrated terminal nucleotidyl transferase (TNTase) activity for hPBV polymerase being the second polymerase among dsRNA viruses reported with TNTase activity. Co-expression of the hPBV RdRP and CP resulted in the formation of ~35 nm VLPs that were incapable of sequestering the RdRP molecules. Results from gel shift assays indicate that the hPBV RdRP has a strong preference for the 5’-terminal untranslated region of the positive-sense genomic RNA (*i*.*e*. 5’(+) UTR). Our results thus suggest that PBV most likely has its RdRP molecules incorporated into viral particles through direct interactions with the genomic RNAs, which are selectively packaged through specific interactions with the viral CP.

## Results

### Overview of the PBV RdRP Structure

**Fig 1:**
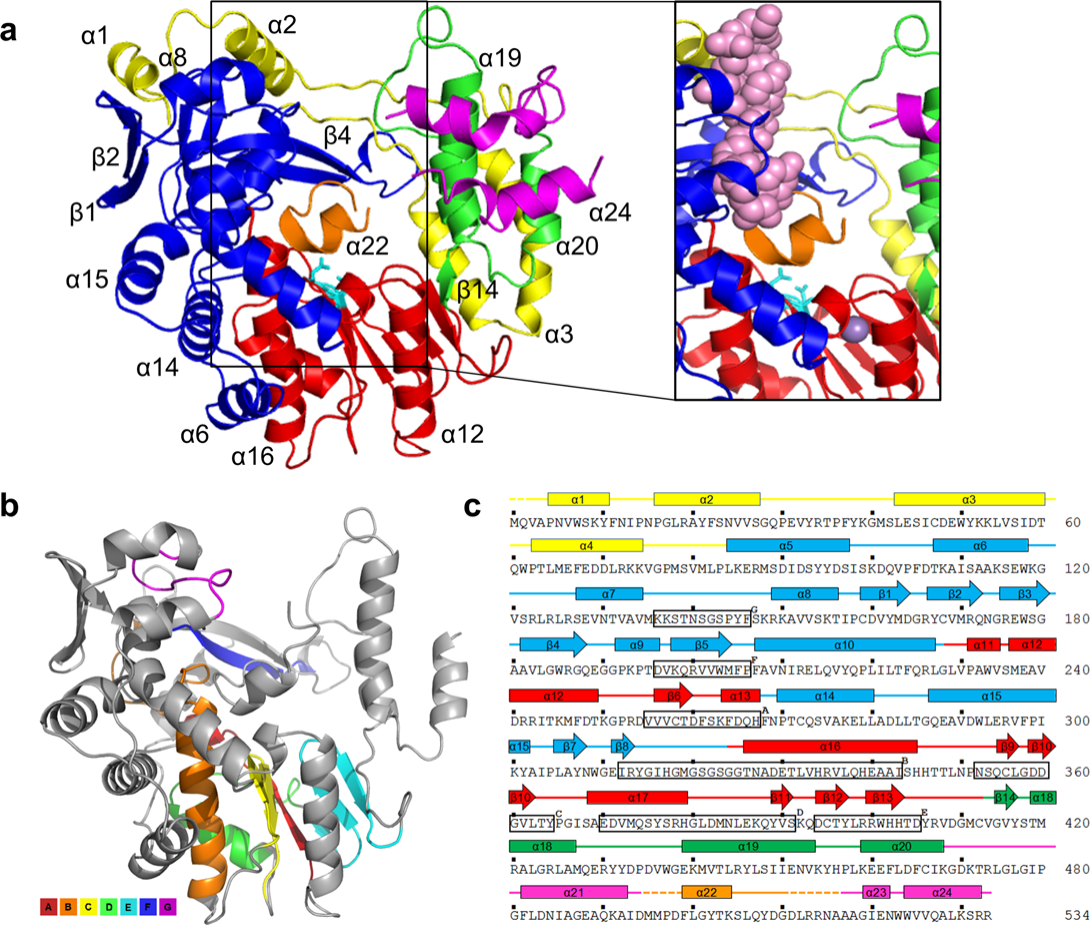
The structure of the hPBV RdRP. (a) Ribbon diagram of the hPBV RdRP crystal structure. The N- and C-terminal domains are colored in yellow and magenta, respectively. The core polymerase domain is shown in three different colors with the *fingers* in blue, the *palm* in red, and the *thumb* subdomain in green. The three key aspartic acid residues are displayed in teal and the flexible insertion loop structure is highlighted in orange. Additionally, a close-up is shown of the hPBV RdRP superimposed with the surface view of the oligonucleotide from the ϕ6 RdRP replication initiation complex (PDB ID 1HI0). The oligonucleotide is colored in pink and the Mn^2+^ ion is colored grey. (b) The seven conserved core polymerase domain motifs. The N- and C-terminal domains are omitted for clarity. Different motifs are colored according to the color keys shown in the figure. (c) Secondary structure assignment of the hPBV RdRP. Disordered regions are shown by dashed lines. α-helices and β-strands are represented by cylinders and arrows, respectively. The seven polymerase motifs are boxed and labeled sequentially as G, F, A, B, C, D, and E. The color scheme is the same as in (a).

Recombinant hPBV RdRP (strain Hy005102) was overexpressed in *Escherichia coli* as a soluble protein. The purified hPBV RdRP (534 aa, ~62 kDa with a N-terminal 6xHis tag) exists in solution as a monomer, based on its gel filtration chromatography elution profile (S1 Fig). hPBV RdRP was crystallized in the space group of P2_1_ with two molecules in each asymmetric unit. The crystal structure was solved to 2.4 Å resolution by single-wavelength anomalous dispersion (SAD) using Se-Met derivatized crystals (Table 1). In the final model of the hPBV RdRP, one molecule is comprised of residues 1–494, 500–511, and 518–534 (Fig 1A), while the other molecule contains residues 1–495 and 520–534.

**Table 1:** PBV RdRP data collection and refinement statistics.

	Apo WT RdRP ^1^	ΔLOOP RdRP ^1^
**Structure determination**		
Space Group	P2_1_	P4_1_2_1_2
Unite Cell Dimensions, Å	a = 75.7, b = 78.8, c = 101.8, β = 91.4°	a = 77.5, c = 183.8
Resolution, Å	50–2.4	50–2.0
Number of frames	180	180
Number of reflections	165,949	505,891
Completeness	99.4% (96.5%)	99.9% (98.8%)
I/σ	10.4 (2.4)	19.6 (2.5)
R_merge_	0.11 (0.369)	0.093 (0.235)
Wavelength, Å	0.979	0.979
Molecules per Asymmetric Unit	2	1
SeMet Sites	36	n/a
**Refinement**		
R_free_	0.224	0.201
R_work_	0.170	0.172
Ramachandran Favored	96.8%	98.6%
RMS of bond lengths and angels	0.010Å, 1.080°	0.008Å, 0.900°

^1^ The numbers in parenthesis are for the highest resolution shell

**Fig 2:**
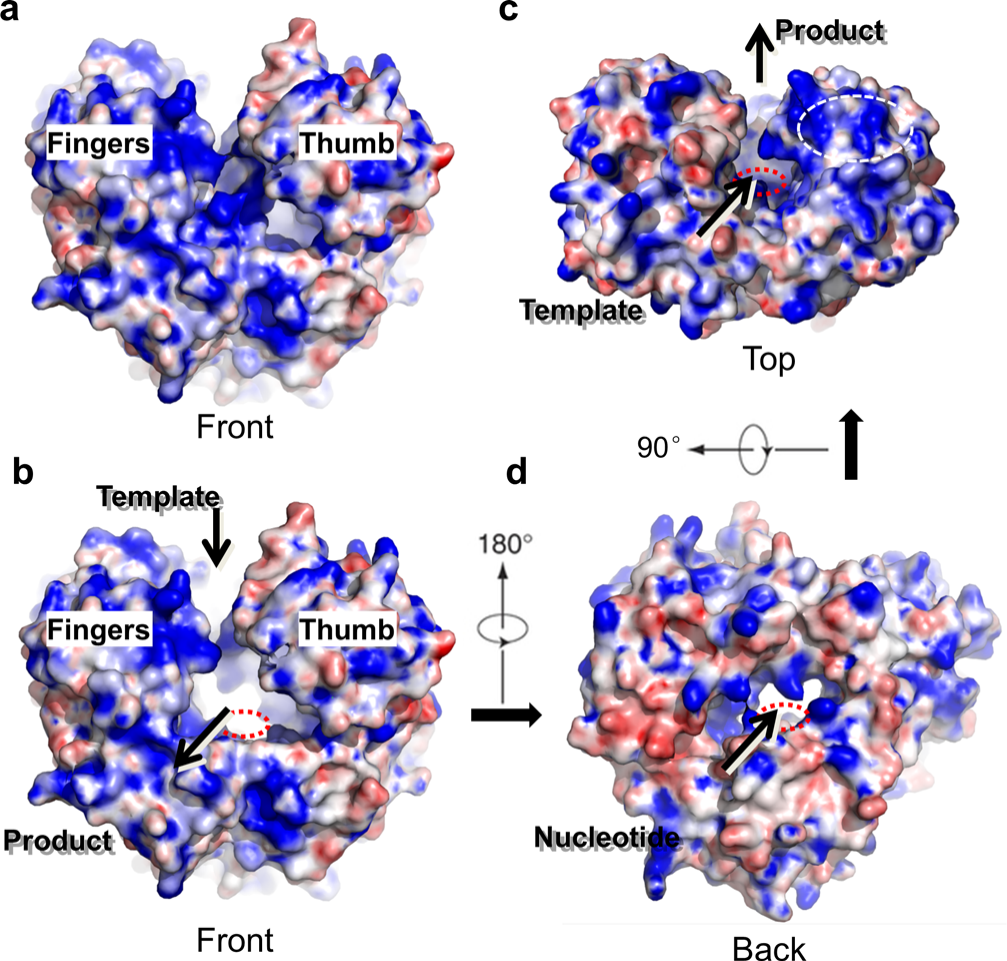
Surface representation of the hPBV RdRP. (a) The WT RdRP molecule is shown from the front end in a similar orientation as in Fig 1A and colored according to its electrostatic potential with positively charged regions in blue and negatively charged regions in red. (b-d) WT RdRP with the insertion loop removed to reveal channels connected to the active site. Three consecutive views are provided, front (b), top (c) and back (d), that show the product channel, the template entry channel, and the nucleotide diffusion channel, respectively. In the top view (c), a positively charged groove next to the template entry channel is highlighted by a white dotted oval. The red dotted circle illustrates the approximate position of the polymerase active site in (b-d).

The hPBV RdRP has an overall oval shape and is ~50 x 60 x 60 Å^3^ in size (Figs 1 and 2). There are a total of 24 α-helices and 14 β-strands in each molecule. The polypeptide can be divided into three domains based on their function: an N-terminal domain (aa 1–84), a core polymerase domain (aa 85–470), and a C-terminal domain (aa 471–534) (Fig 1A and 1C; S2 Fig). The polymerase domain, which is structurally highly conserved amongst RNA viruses [39], has a right-hand configuration with three subdomains: the *fingers* (aa 85–230, 268–324), *palm* (aa 231–267, 325–414), and *thumb* (aa 415–470) (Fig 1A and 1C). The *palm* subdomain hosts the three key aspartic acid residues, D261, D359, and D360, of the active site (Fig 1A).

### Domain Structure of the hPBV RdRP

The hPBV core polymerase domain is rather compact with only 386 aa in total (Fig 1B). The *palm* subdomain, which hosts the catalytic active site, is composed of five α-helices and six β-strands (*i*.*e*. α11–13, 16, 17 and β6, 9–13) and contains the polymerase motifs A-E that are conserved among all RdRPs (Fig 1B and 1C; red) [40]. The most noticeable structural feature of the *palm* is a central, four-stranded β-sheet consisting of a β-hairpin (*i*.*e*. β9, β10) and two anti-parallel β-strands (*i*.*e*. β6, β11). The polymerase motif C, which contains the highly conserved “-GDD-” sequence, is mapped to the β-hairpin. The two other β-strands (β6, β11) of the central β-sheet contain motifs A and D, respectively. The motif A has the conserved sequence of “DXXXXD” whereas motif D is more variable in sequence. The first aspartate from motif A (*i*.*e*. D261) and the two aspartates from motif C (*i*.*e*. D359 and D360) constitute the active site as they help to coordinate two divalent metal ions for charge relay and intermediate stabilization during catalysis. The motif D mediates the binding of the incoming nucleotide substrate and plays an important role in determining the efficiency and fidelity of nucleotide addition [41]. Motif B, which folds into a strand-turn-helix structure at the interface between the *fingers* and *palm* subdomains, has been found to interact with the RNA template to guide it into the active site of the polymerase [42]. Motif E folds into a β-hairpin at the interface between the *palm* and the *thumb* subdomains, and forms a part of the “primer grip” as discussed below [40].

Close inspection shows that the *fingers* subdomain of the hPBV RdRP is composed of eight α-helices and seven β-strands (Fig 1A and 1C; blue). At the top of the *fingers* subdomain is a twisted, four-stranded β-sheet (β4, β5, β7, and β8) that forms the *fingertip* structure with an extended loop, which contains the rNTP binding sequence (residues 182–199) denoted as the RdRP motif F (Fig 1B and 1C) [40]. Structural studies on ϕ6 and hepatitis C virus (HCV) RdRPs have revealed that the basic residues in the rNTP binding loop interact with the phosphates of the incoming nucleotide [18,43]. The *fingers* subdomain also contains the RdRP motif G [44] that is located near a three-stranded antiparallel β-sheet (β1, β2, and β3; Fig 1B and 1C). The structures of the ϕ6 and reovirus RdRPs have revealed that the residues of motif G interact with the entering RNA template [18,21].

Situated at the other side of the polymerase *palm* across from the *fingers* subdomain is the *thumb* subdomain, which is comprised of one β-strand (β14) followed by three α-helices (α18 - α20; Fig 1A and 1C; green). The initial β14-strand forms a part of the “primer grip” along with a β-hairpin (β12 and β13) from the *palm* subdomain (Fig 1A). The “primer grip” motif is commonly observed in viral RdRPs and has been shown to interact with the nascent/primer strand during RNA synthesis [40].

The hPBV N-terminal domain is made of the first 84 residues of the polypeptide and contains four α-helices (α1–4). With an overall L-shape, the N-terminal domain wraps around the *fingers* and *thumb* subdomains with its long and short arms, respectively (Fig 1A; yellow). This interaction allows the RdRP to maintain its active site in a closed conformation despite that there is very little direct contact between the hPBV fingertip and the *thumb* subdomain. The N-terminal domain in the RdRPs from infectious bursal disease virus, reovirus, and the rabbit hemorrhagic disease viruses also helps to encircle the polymerase active site although the size of these N-terminal domains can vary substantially [20,21,45]. The C-terminal domain of hPBV is rather short with four α-helices. It lies adjacent to the *thumb* subdomain at the front end of the polymerase *palm* (Fig 1A; magenta).

The hPBV RdRP contains three channels leading to the active site of the protein that are believed to allow for dsRNA product export, template entry and NTP uptake (Fig 2). The dsRNA product channel, which locates in the front of the molecule, is the largest of all three with a diameter of ~18–20 Å, comparable to the diameter of a dsRNA helix (Fig 2A–2C). Both the template and NTP channels are located near the interface between the *fingers* and *thumb* subdomains, and are heavily lined with positively charged residues (Fig 2B, 2C and 2D). The distance from the surface of the RdRP to the active site along the putative template entry channel could be spanned by 5 to 6 nucleotides as observed in the ϕ6 RdRP [46]. A patch of positively charged residues is found near the putative template entry channel (Fig 2C, dotted oval). A positively charged plough has been previously noted in the structure of ϕ6 RdRP, and is believed to play a role in separating the two strands of a dsRNA molecule allowing the template RNA to enter the template entry channel, while the non-template RNA slides over the positively charged patch and is directed away from the RdRP [18].

Based upon a pairwise comparison using the program Dali [47], the structure of the hPBV RdRP closely resembles that of the RdRPs from the members of the *Caliciviridae* (*i*.*e*. Z = 26.7 for the rabbit hemorrhagic disease virus and Z = 26.5 for the Norwalk virus) [45,48], *Flaviviridae* (*i*.*e*. Z = 26.4 for the HCV and Z = 24.2 for the bovine viral diarrhea virus (BVDV)) [43,49–51] and *Picornaviridae* (*i*.*e*. Z = 25.3 for the poliovirus) [52] families, suggesting a potential evolutionary relationship between the RdRP of PBV and the RdRPs of these three viral families having positive-sense ssRNA genomes. By contrast, the correlation of the hPBV polymerase to the RdRPs from other dsRNA virus families appears to be more distant, with a Z = 18.8 for phage ϕ6 [18], Z = 12.8 for rotavirus [19], Z = 12.1 for infectious bursal disease virus [20], and Z = 9.3 for reovirus [21].

### Insertion Loop Structure

The hPBV RdRP possesses a highly flexible, 24-aa insertion loop structure (aa 495–518) formed by an internal sequence from the C-terminal domain. This loop structure, which is associated with higher than average temperature factor values, extends from the C-terminal domain towards the catalytic site (Fig 1A). The location of the insertion loop resembles the structure that functions as the initiation platform of the bacteriophage ϕ6 RdRP [23,24]. The structure of the ϕ6 RdRP in complex with an oligonucleotide template (PDB ID 1HI0) was superimposed onto the hPBV RdRP structure in order to model RNA binding by the hPBV RdRP. In this model, the 3’-end of the template collides with the hPBV insertion loop structure, suggesting that the insertion loop in its current position would sterically prevent the template from binding to the active site of the RdRP as expected in the assembly of a productive initiation complex (Fig 1A, right). Therefore, the hPBV insertion loop structure must undergo a significant conformational change in order for RNA binding and replication to take place. Similar structural rearrangement is probably required for dsRNA egress during both transcription and genome replication. Interestingly, such structural arrangements were indeed observed in the β-hairpin structure that serves as an initiation platform for the HCV RdRP following RNA binding [53]. In the ϕ6 polymerase, the loop that serves as the initiation platform remains in place during the assembly of the initiation complex, but undergoes a conformational change at the onset of the elongation process [54].

Based on our structural modeling and comparison to both the ϕ6 and HCV polymerases, we speculate that the insertion loop of the hPBV RdRP may have two possible functions. First, the loop may play an important role during the *de novo* initiation of RNA synthesis. Like in the case of the ϕ6, HCV and several other flavivirus polymerases, the loop could function as a platform to support the assembly of an initiation complex using a single nucleotide as a primer. Another attractive hypothesis is that the insertion loop may function as a gatekeeper to regulate RNA synthesis during the PBV virion assembly. The insertion loop in its observed structural form would inhibit an RNA template from binding, but upon the RdRP binding with the CP (*i*.*e*. in an assembled capsid), the insertion loop would adopt an alternative conformation to allow efficient RNA synthesis in the fully/partially assembled hPBV particles.

### Structure of the PBV RdRP without the Insertion Loop

**Fig 3:**
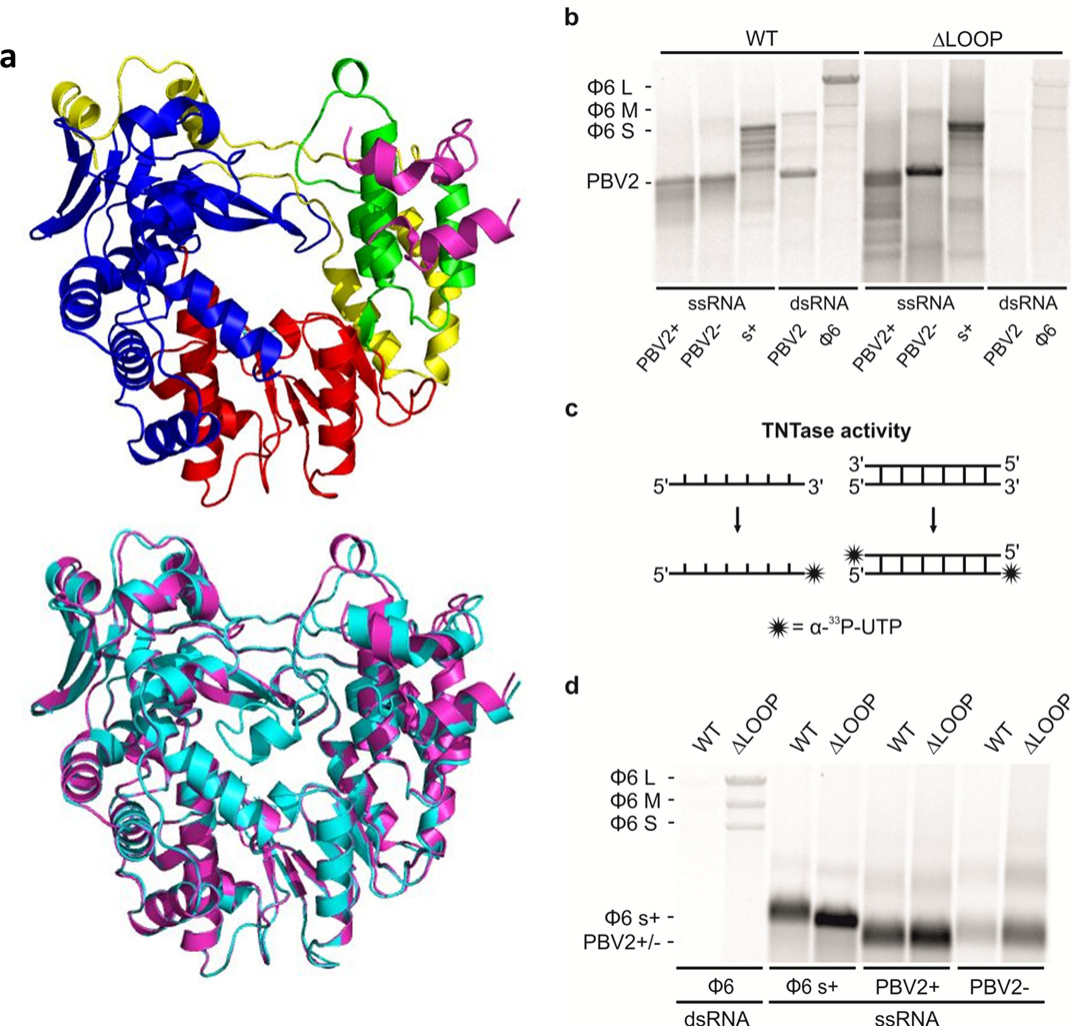
The structure and enzymatic activies of the hPBV WT and ΔLOOP RdRP. (a) Ribbon diagram of the hPBV ΔLOOP RdRP crystal structure. The color scheme and the viewing orientation is the same as that used for the WT RdRP in Fig 1A. Below is a structural alignment between the WT (cyan) and the ΔLOOP (magenta) RdRPs. (b) The replication and transcription activity and the template specificity of the hPBV WT (left) and ΔLOOP (right) RdRPs. Three ssRNA and two dsRNA templates were used: (+) and (-)strands of the PBV genome segment 2 (PBV2+ and PBV-, respectively), (+)strand of the ϕ6 genomic segment S (s+), PBV dsRNA genome segment 2 (PBV2) and ϕ6 genomic dsRNA (ϕ6). (c) A schematic representation of the TNTase activity using either ssRNA (left) or dsRNA (right) as a substrate. (d) TNTase activity assays for the WT and the ΔLOOP RdRPs using either ϕ6 genomic dsRNA (left) or ϕ6 and PBV specific ssRNA substrates (right). The RdRPs used are indicated on the top and the RNA templates on the bottom (b and d). The mobility of the ϕ6 and hPBV-specific dsRNAs and ssRNAs are marked on the left (b and d).

To determine the exact biological function of the insertion loop structure, a hPBV RdRP lacking the loop structure (*i*.*e*. ΔLOOP) was synthesized and expressed. Gel filtration chromatography showed that ΔLOOP was eluted at a similar position as the WT protein (S1 Fig). The ΔLOOP RdRP was crystallized and its structure solved by molecular replacement using the WT RdRP structure as the phasing model (Fig 3A, Table 1). The overall structure of ΔLOOP appears to be essentially the same as the WT RdRP (root-mean-square deviation in distance = 0.4 Å for 3275 common atoms), except for the deleted insertion loop which became unstructured in the ΔLOOP RdRP. Given this structural information, we are confident that the removal of the insertion loop should not affect the overall folding of the polymerase, and that the ΔLOOP RdRP should provide an excellent tool to study the function of the insertion loop structure using *in vitro* assays.

### Enzymatic Activities of the WT and ΔLOOP RdRPs

Polymerase activity assays were performed to determine if the WT and ΔLOOP RdRPs could synthesize dsRNA from an ssRNA template. Both RdRPs were found to replicate the positive- and negative-strands of the PBV genome segment 2 (PBV2+ and PBV2-, respectively) as well as ϕ6-specific ssRNA template s^+^ (*i*.*e*. the positive-strand of the small, S, genome segment) with similar efficiency (Fig 3B). However, the polymerase encountered some processivity issues, as it produced also dsRNA products which were shorter than the expected full length dsRNAs (Fig 3B). Further enzymatic assays showed that the WT RdRP is able to use dsRNA templates to carry out transcription but the transcription activity of the ΔLOOP RdRP was compromised (Fig 3B). Transcription activity was observed whenever homologous (*i*.*e*. PBV2) or heterologous dsRNAs (*i*.*e*. ϕ6 genomic RNA composed of segments S, M and L) were used as templates (Fig 3B, S1 Table). This indicates that the hPBV RdRP is enzymatically active, can efficiently replicate and transcribe both homologous and heterologous templates, and does not require the presence of the viral CP as a cofactor for RNA synthesis. Our findings thus rule out the scenario where the loop structure functions as a regulatory element to prevent premature dsRNA synthesis by the capsid-free polymerase.

Terminal nucleotidyl transferase (TNTase) activity was observed for both the WT and ΔLOOP RdRPs (Fig 3C and 3D). TNTase activity involves the addition of one or several nucleotide(s) to the 3’-end of a nucleic acid molecule (Fig 3C). While there was no clear preference for the hPBV-specific RNA substrate, both the WT and ΔLOOP RdRPs showed a strong preference for ssRNA over dsRNA (Fig 3D). Additionally, it was noted that the removal of the insertion loop significantly increased the TNTase activity of the RdRP (Fig 3D). Given our TNTase activity data and the fact that the insertion loop is located near the C-terminus of the hPBV RdRP, we propose that the removal of the insertion loop structure leaves the dsRNA exit channel of the protein permanently open (Fig 2B), thus allowing the 3’-end of the RNA molecules to reach the active site for nucleotidyl addition in an orientation that is compatible for nucleotide addition.

To rule out any histidine-tag (His-tag) associated artifacts, three versions of the hPBV RdRP, an N-terminally and C-terminally His-tagged as well as a non-tagged RdRP, were tested for RNA synthesis activity using the PBV2+ and PBV2- ssRNA templates as well as the PBV2 dsRNA template (S3 Fig). Our results indicated that there was no detectable difference in the enzymatic activity between these three versions of the polymerase. Therefore, all of the polymerase activity assays described in this paper has been performed using the N-terminally His-tagged protein.

### The Role of the Insertion Loop in the *De Novo* Initiation

**Fig 4:**
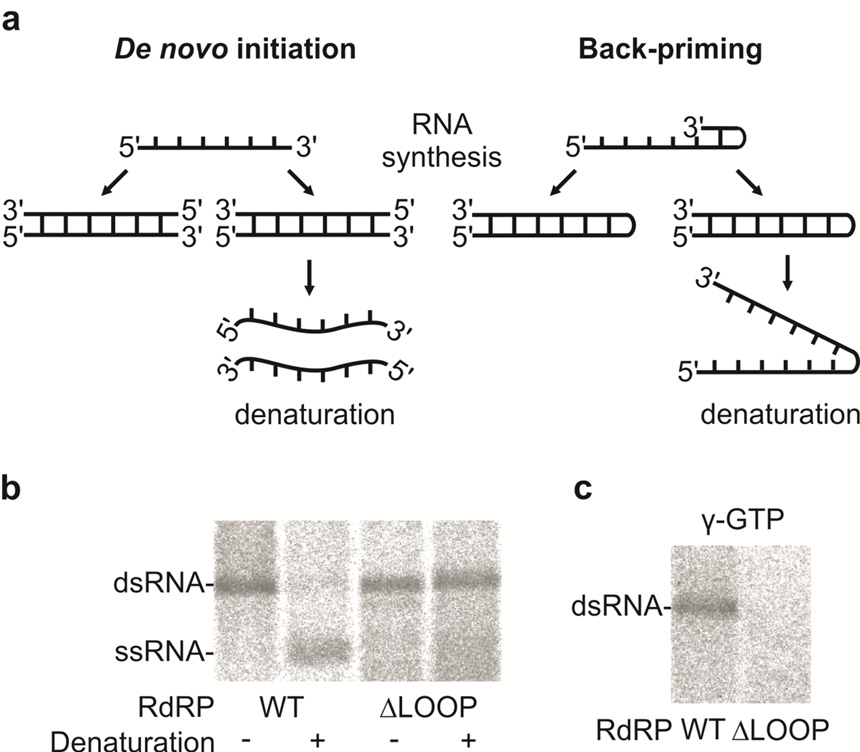
The hPBV RdRP insertion loop enables primer-independent RNA synthesis. (a) A schematic representation of the *de novo* (left) and back-priming (right) initiation modes of the PBV RdRP displaying the effect of the heat-denaturation on the replication reaction products. (b) Native agarose gel electrophoresis of the replication reaction products of ϕ6 Δs^+^ ssRNA before and after denaturation as indicated below. (c) Labeling of the replication reaction products of ϕ6 Δs^+^ ssRNA with [γ-^32^P]-GTP in the initiation of RNA replication. The RdRPs applied are indicated at the bottom and the mobilities of the dsRNAs and ssRNAs are marked on the left (b and c).

To evaluate the potential role of the insertion loop in the initiation of dsRNA synthesis (*i*.*e*. replication), aliquots of the dsRNA products were heat denatured before being analyzed by electrophoresis in a native agarose gel (Fig 4A and 4B). Due to the heat denaturation, the dsRNA products generated by the WT RdRP were converted to ssRNA, while the dsRNA products of the ΔLOOP RdRP retained the same mobility as the original dsRNA (Fig 4A and 4B). This result indicates that back-priming was taking place during dsRNA synthesis by the ΔLOOP RdRP, thus producing a product that was covalently linked to the ssRNA template (Fig 4A, right panel and b). Additionally, isotope incorporation from (γ-^32^P) GTP into the dsRNA product was only detected for reactions containing the WT RdRP, further indicating that this protein utilizes the *de novo* initiation mechanism (Fig 4C). Taken together, these results show that the insertion loop structure of the hPBV RdRP can effectively block template back-priming and facilitates initiation via a primer-independent mechanism, possibly by providing a docking site for the 3’-end of the RNA template and a binding site for the priming nucleotide. This finding is consistent with the results obtained for the ϕ6 RdRP whenever its equivalent stabilizing platform was removed [23,24].

### Semi-conservative Transcription by the hPBV RdRP

**Fig 5:**
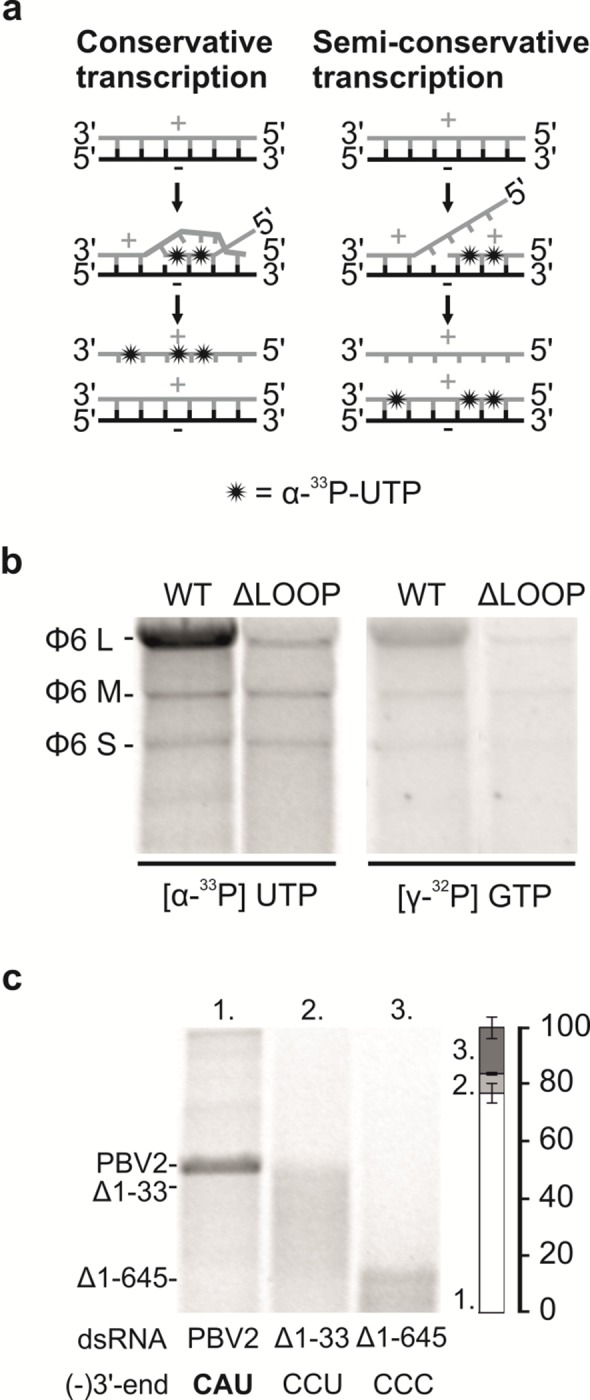
Transcription activity of the hPBV RdRP. (a) Schematic drawings showing radiolabeled products expected from conservative (left) and semi-conservative transcription (right). (b) Transcription activity of the WT and the ΔLOOP RdRPs using virion derived ϕ6 genomic dsRNA. The expected position of the L, M, S dsRNA segments are indicated on the left. Both [α-^33^P] UTP (left) and [ү-^32^P] GTP (right) were used to label reaction products. The RdRPs applied are indicated at the top. (c) Transcription activity of the WT RdRPs using PBV2 dsRNA templates synthesized *in vitro*. Three dsRNA templates were included: the full-length PBV2 (*i*.*e*. PBV2), PBV2 without the first 33 base pairs (*i*.*e*. Δ1–33), and PBV2 without the first 645 base pairs (*i*.*e*. Δ1–645). For the quantitation (right), the gel band intensities were normalized by the length (bp) of the dsRNA molecules. The RNAs applied and their (-)strand 3’-end sequences are shown at the bottom. The mobilities of the dsRNAs are marked on the left (b and c).

There are two major ways for the transcription of the dsRNA genome: conservative and semi-conservative transcription mechanism (Fig 5A). The isotope labeling of the dsRNA molecules as an outcome of the transcription reaction indicated that the hPBV polymerase uses a semi-conservative transcription mechanism (Figs 3B, 5A and 5B). The ability of the hPBV RdRP to incorporate radioactivity from [γ-^32^P]-labeled GTP into the dsRNA product also confirmed that the observation of radiolabeled dsRNAs was due to de novo RNA synthesis instead of terminal nucleotidyl transfer catalyzed by the polymerase (Fig 5B, right panel). To get further support for the semi-conservative mechanism a time-course study using the ϕ6 genomic dsRNA as a template was performed (S4 Fig). Indeed, ^33^P-label was first incorporated in dsRNA molecules before any labeled ssRNA could be observed (S4 Fig), confirming that semi-conservative transcription was taken place (not replication of ssRNA transcripts produced by conservative mechanism). The ϕ6 L segment was transcribed at the highest efficiency, resulting in a strong dsRNA band that started to appear at around 30 minutes with increasing intensity till 120 minutes after the experiment began (S4 Fig). Considering that the L segment of ϕ6 is ~6.4kb long, we estimate that the rate of transcription by the hPBV RdRP is ~210 bases per minute.

### Template- and Strand-Specificity of the hPBV RdRP

The (-)strands of the two hPBV genome segments both start with 3’-CAU (S1 Table), so we set forth to test the transcription specificity of the hPBV RdRP using dsRNA template with different terminal sequences. Coincidentally, the (-)strand of the ϕ6 L segment also starts with 3’-CAU, while the (-)strands of the ϕ6 S and M segments begin with 3’-CCU (S1 Table). We found that, under the applied reaction conditions, the hPBV RdRP has a preference for dsRNA templates in which the (-)strand starts with 3’-CAU over those starting with 3’-CCU (S4 Fig, Figs 3B and 5B). The preference for the ϕ6 L segment over the M and S segments was lost when the terminal sequence of L was mutated to 3’-CCU (S5 Fig). On the other hand, when an opposite change was introduced in the (-)strands of the ϕ6 genome segments M and S (3’-CCU to 3’-CAU), the amount of product synthesized by the hPBV RdRP increased considerably (S5 Fig). The template preference of the hPBV RdRP was also evident when the usage of the PBV2 and ϕ6 S or ϕ6 L dsRNAs was compared in a competition experiment or side-by-side (S6B Fig).

We then tested the ability of hPBV RdRP to transcribe full-length PBV2 and its two deletion mutants, one with the first 33 nucleotides removed and the other with the first 645 nucleotides removed (Fig 5C). As a result of the deletions, the 3’-ends of the (-) strands of the two truncation mutants, Δ1–33 and Δ1–645, became 3’-CCU and 3’-CCC, respectively. Transcription assays showed that out of the three dsRNA templates, only the WT PBV2, which has a 3’-CAU termini at the (-)strand, was efficiently transcribed (Fig 5C). Taking together the results with the PBV2- and ϕ6-specific dsRNA constructs (S5 Fig and Fig 5C) sharing identical 3’-terminal sequences in (+)strands (S1 Table) but variable in (-)strands, it appears that PBV RdRP has strong preference on dsRNA templates having 3’-CAU. This data also suggest that the RdRP predominantly produces (+)strands of the PBV2 and ϕ6 genome segments during transcription reaction.

There are two possible explanations for the stronger transcription activity exhibited by the hPBV RdRP towards native PBV2 (-)strand sequence ending with 3’-CAU. First, the lower melting temperature associated with the 3’-CAU sequence likely facilitates the initiation of RNA transcription. Second, it is possible that the 3’-CAU sequence makes specific interaction with the hPBV RdRP, which would facilitate the binding of the viral RNA template and thus helps to enhance its transcription activity. We have not observed any preference of the hPBV RdRP towards ssRNA template (Figs 3B and S6A). Indeed, the fact that virus-specific ssRNA was not replicated to a higher level when mixed with non-specific ssRNA in a competition experiment argues against the second hypothesis. Therefore, we believe that the preference for virus-specific dsRNA templates by the hPBV RdRP during transcription is largely due to the lower melting temperature of the template terminal sequence.

### RdRP Encapsidation during hPBV Assembly

**Fig 6:**
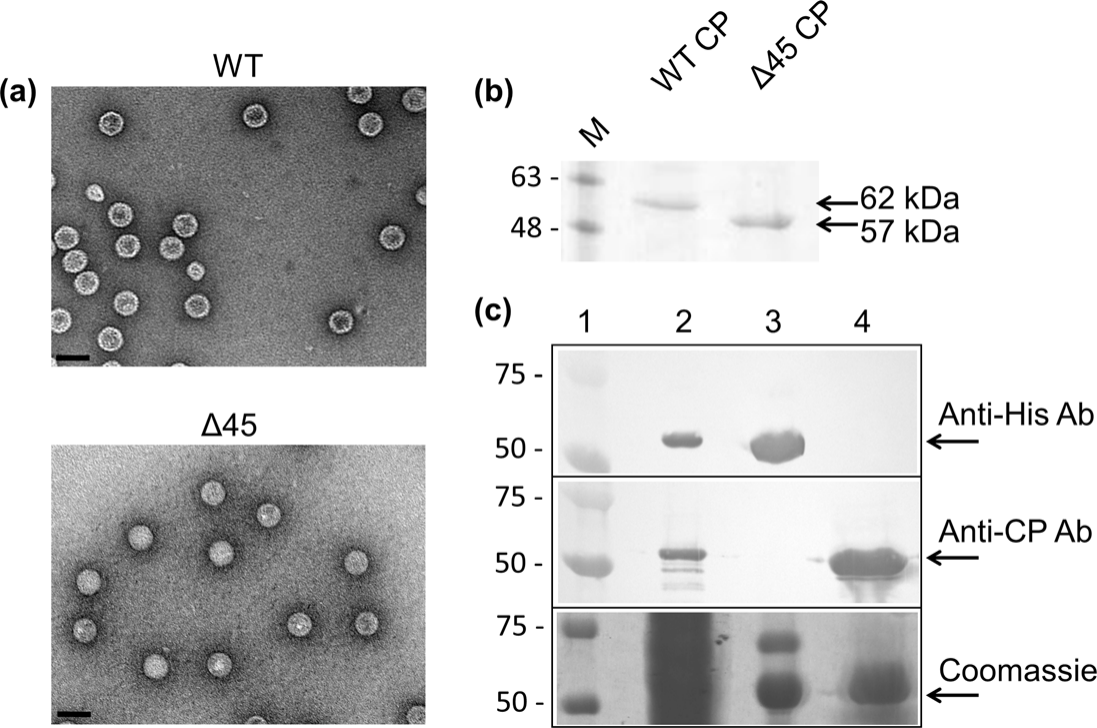
Co-expression of the hPBV RdRP and CP. (a) TEM images of the purified hPBV VLPs for the WT CP (top) and the Δ45CP (bottom). The scale bar represents 50 nm. (b) SDS-PAGE analysis of the recombinant capsids of both the full-length and Δ45 CPs. (c) Co-expression of the hPBV CP and RdRP. Samples included are a prestained protein marker (lane 1), the soluble fraction of the cell lysate (lane 2), the Ni-NTA bound fraction (lane 3), and the purified VLPs (lane 4). Proteins were separated by SDS-PAGE and detected by Western blot using either anti-6xHis or anti-CP antibodies (two upper panels) or staining with Coomassie blue (lower panel). The molecular weights of the pre-stained marker proteins are indicated in kDa on the left.

Upon finding that the hPBV RdRP was capable of synthesizing dsRNA and ssRNA in the absence of the CP (Fig 3B), the hPBV RdRP and CP were co-expressed to determine if the encapsidation of the RdRP is mediated through direct protein-protein interactions with the CP, as has been previously observed for the majority of dsRNA viruses characterized so far [6,8,9]. Overexpression of the hPBV CP in *E*. *coli* resulted in the spontaneous formation of VLPs. Such VLPs could be purified by ultracentrifugation using a CsCl gradient and have a density of ~1.3 g/ml according to their migration behavior. Transmission electron microscopy (TEM) images of the hPBV VLPs determined that they have a diameter of ~35 nm (Fig 6A), similar to the size of the rabbit PBV VLPs previously reported [34]. While the N-terminus of the rabbit PBV CP is proteolytically removed by self-cleavage after particle assembly, this does not seem to be the case for the hPBV CP. We found that the hPBV CP without the N-terminal peptide (Δ45) was also capable of self-assembly, and that the N-terminally truncated CP migrated faster than the full-length protein in a reducing sodium dodecyl sulfate polyacrylamide gel electrophoresis (SDS-PAGE) (Fig 6A and 6B).

The hPBV RdRP and the CP were co-expressed in *E*. *coli* using two expression vectors with different antibiotic selectors. Any unpackaged His-tagged RdRP molecules that may have been present in the clarified cell lysate were removed by Ni-NTA affinity chromatography. After ultracentrifugation, the VLP fraction was collected, denatured, and a Western blot was performed to test for the presence of the RdRP utilizing an antibody directed against the 6xHis-tag located at the N-terminus of the protein (Fig 6C). While the RdRP could be clearly detected in the clarified lysate, no RdRP could be detected in the sample of the isolated VLPs. Therefore, our results suggest that direct interactions between the hPBV RdRP and CP are weak. We hypothesize that viral genomic material must be present for RdRP incorporation to occur and that the interplay between the CP, RdRP, and the viral genome is needed for the encapsidation of the RdRP during virion assembly. The fact that the constructs used to express the CP and RdRP contained only the protein-coding sequence of the two open reading frames suggests that the region of the hPBV genome required for RdRP encapsidation is potentially located in the untranslated regions (UTRs) of the genome segments.

### Recognition of the hPBV Genome by the RdRP

**Fig 7:**
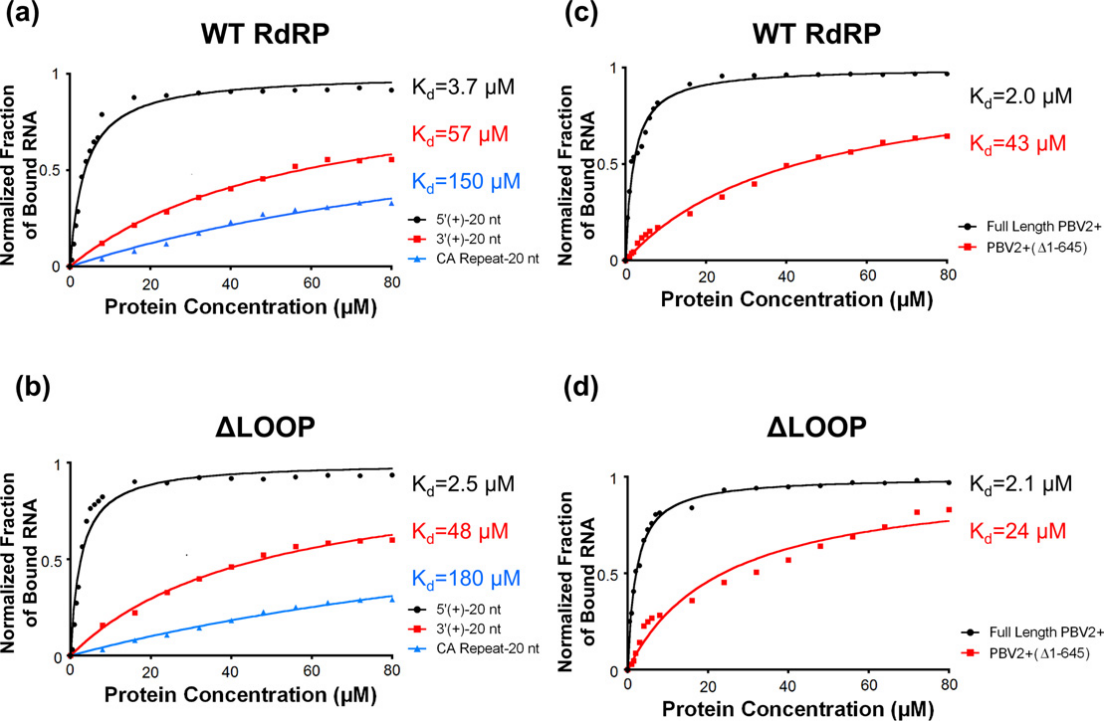
RNA binding by the hPBV WT (a, c) and ΔLOOP (b, d) RdRPs analyzed by gel shift assays. The fraction of RNA bound was quantitated and plotted as a function of protein concentration. The values were normalized by the total amount of RNA. (a, c) Three distinct RNA oligonucleotides were used, including: (1) the first 20 nucleotides of the 5’-(+)UTR of hPBV genome segment 2 (black); (2) the last 20 nucleotides of the 3’-(+)UTR of the hPBV genome segment 2 (red); and (3) a 20-mer nonsensical CA repeat (blue). (b, d) Two RNA molecules derived from the PBV2+ were used, including the full-length PBV2+ and the PBV2+(Δ1–645). The obtained dissociation constants (K_d_) for the different RNA oligonucleotides are indicated on the right.

Gel shift assays were conducted to further examine the interaction between the RdRP and the hPBV genome. Three 20-nt long RNA oligonucleotides were probed, one containing a non-specific CA-repeat and the other two bearing the terminal sequences of the 5’- and 3’-UTRs of the (+)strand of the hPBV genome segment 2 (*i*.*e*. 5’-(+)UTR and 3’-(+)UTR). It was determined that the WT RdRP has approximately a 10-fold higher affinity for the first 20 nucleotides of the 5’-(+)UTR of the hPBV genome as compared to the last 20 nucleotides of the 3’-(+)UTR or to a nonsensical CA-repeat (Fig 7A). Additionally, deleting the insertion loop appeared to have a minimal effect on the overall RNA binding of the RdRP (Fig 7B).

Notably, both segments of the hPBV genome have an AU-rich (~80% for nucleotides 1 to 20) sequence at the 5’-end of their (+)strands that begins with a conserved 5 nucleotide motif 5’-GUAAA-. The AU-rich terminal sequence is predicted to form an RNA stem-loop structure (S7 Fig) according to the program ViennaRNA Package 2.0 [55]. To test the binding affinity of the hPBV RdRP towards authentic viral RNAs, we synthesized by *in vitro* transcription the full-length PBV2+ ssRNA and a truncated version PBV2+ (Δ1–645) ssRNA with the first 645 nucleotides removed. Results from our gel shift assays show that the binding affinity of hPBV RdRP for full-length PBV2+ is ~10 times stronger than PBV2+(Δ1–645), thus confirming that the terminal sequence at the 5’-end of the PBV2+ binds specifically to the polymerase (Fig 7C). The same trend was observed for the ΔLOOP RdRP, suggesting that the insertion loop is not directly involved in the 5’-(+)UTR binding (Fig 7D).

## Discussion

The high level of sequence conservation among RdRPs from various mammalian and avian PBV strains indicates similar three-dimensional structures (S2A Fig). Five of the six variable regions are located in either the N-terminal domain or the *fingers* subdomain, and all are mapped to the surface of the protein (S2B Fig). The core polymerase domain of hPBV RdRP closely resembles those from the members of the *Caliciviridae*, *Flaviviridae*, *and Picornaviridae* families all having (+)sense ssRNA genomes. An interesting structural feature of this RdRP is the presence of a 24-aa loop structure that extends from near the C-terminus of the protein to just above the active site (Fig 1A). Through site-directed mutagenesis, we determined that the loop structure most likely functions as a priming platform to support the binding of a single priming nucleotide (Fig 4). Loop structures that perform a similar function have been observed in the viral RdRPs of ϕ6, reovirus, rotavirus, and HCV [18,19,21,23,24,53]. In four-tunnel RdRPs such as those from reovirus and rotavirus, the internal priming loops are formed by sequences located between the fingers and palm domain. However, in three-tunnel polymerases that catalyze semi-conservative RNA synthesis (i.e. ϕ6 and HCV), the priming loops appear to be extended structures from the C-terminal domain. The position of the insertion loop in the hPBV *apo* structure would prevent a template from reaching the active site due to steric hindrance (Fig 1A, right panel). Therefore, we expect the insertion loop to undergo a significant conformational change in order to accommodate an RNA template, similar to the conformational change observed for the HCV priming loop upon template binding [53]. While the first half of the insertion loop sequence is highly conserved, the other half is somewhat variable (S2B Fig). We speculate that the conserved region, including a strictly conserved tyrosine, may interact with the priming nucleotide and/or template to support initiation.

Structural elements that support priming by a single nucleotide are also known to prevent back-priming by spatially restricting access to the active site. Back-priming occurs during RNA synthesis when the 3’-end of the template strand loops back to form a hairpin like structure that is then extended by the RdRP [23] (Fig 4A, right panel). This results in the daughter strand being covalently linked to the initial template preventing further replication of the back-primed RNA. This phenomenon has been observed *in vitro* for HCV, BVDV, and a ϕ6 RdRP lacking the priming loop [23,24,56–59]. Likewise, our results indicate that the insertion loop structure from the hPBV RdRP can effectively prevent back-priming during dsRNA synthesis (Fig 4B) and to support the *de novo* initiation (Fig 4C), consistent with the expected functionality for the priming loop based on previous observations in other RdRPs.

In this paper we have systematically characterized the replication and transcription activity of the hPBV RdRP as a paradigm for the *Picobirnaviridae* family. *In vitro*, the hPBV RdRP is able to catalyze RNA synthesis using both ssRNA and dsRNA templates in the absence of the viral CP (Figs 3 and 5). The enhanced transcription activity observed for the WT protein using hPBV-specific dsRNA or templates harboring homologous 5’ terminal sequences (Fig 5C and S6 Fig) can be explained by the lower melting temperature associated with the terminal sequences. Alternatively, the enhanced transcriptional activity of the hPBV specific dsRNA may be explained by base-specific interaction between the template and the RdRP itself, but we consider it unlikely because enhanced replication activities were not observed for hPBV-specific ssRNA (Fig 3B). Interestingly, the identity of the second nucleotide of the template RNA also regulates the transcription activity of the phage ϕ6 RdRP [60].

hPBV RdRP appears to transcribe dsRNA templates in a semi-conservative fashion (Fig 5). Results from our time-course study show that nucleotides labeled with α-^33^P were first incorporated into dsRNA, indicating that the newly synthesized RNA formed a duplex RNA with its template RNA (S4 Fig; Fig 5A, right panel). Except for the RdRPs of the members of *Reoviridae* family, semi-conservative transcription is reported for most of the dsRNA virus polymerases characterized to-date, including RdRPs from the members of *Partitiviridae* [61], *Birnaviridae* [62], and *Cystoviridae* [63] families. The rate of transcription by hPBV RdRP is ~210 bases per minute. Although we have not experimentally confirmed that the (-)strand is used as a template for transcription, our results with the terminally mutated PBV- and ϕ6-specific dsRNAs strongly favor a scenario in which the sequence at the 3’-end of the (-)strand rather than (+)strand determines the transcription efficiency (Figs 5C and S5, S1 Table). This likely reflects the substantially lower melting temperature associated with the 3’-end of the (-)strands compared to that of the (+)strands (S1 Table).

We also observed TNTase activity for both the WT and ΔLOOP hPBV RdRPs (Fig 3C and 3D). TNTase activity involves the addition of one or several, non-templated nucleotide(s) to the 3’-end of an RNA molecule, and has previously been observed for a number of RdRPs including those from HCV, BVDV, norovirus, poliovirus, and ϕ6 [64–67]. For many viral RdRPs the biological implication of the TNTase activity is not yet clear. Template-independent TNTase activity is probably used by the RdRP as a mechanism to terminate the synthesis of nascent RNA strands, which would acquire one or more extra nucleotides at the 3’-end [65]. Alternatively, TNTase activity may function to repair the 3’-ends of the viral genomes that have been partially degraded [66]. The results of our experiments support the notion that the RNA substrate for the TNTase activity potentially enters the active site through the product exit channel, as removing the insertion loop would leave the product exit channel of the protein permanently open, thus explaining why the ΔLOOP RdRP displayed higher TNTase activity than the WT enzyme, especially for the dsRNA substrates (Fig 3D).

All dsRNA viruses enclose RdRP molecules within their infectious particles. Our results indicate that the hPBV RdRP and CP do not directly interact during the capsid assembly and that the viral RdRP cannot be incorporated into the viral capsid in the absence of the viral genome (Fig 6C). This finding is surprising because several other dsRNA viruses have been found to package their RdRP molecules through direct protein-protein interactions or by expressing a CP-RdRP fusion protein that is then incorporated into the viral particles as a minor structural component [6,8,9,11,12]. We propose that the PBV RdRP molecules get encapsidated as a complex with the viral genomic RNA, as we found that the hPBV polymerase preferably interacts with the 5’-end of the (+)strand. It is likely that the co-packaging mechanism applies not only to PBV but also to partitiviruses, another family of dsRNA viruses with a small capsid that is arranged in a manner similar to PBV [35,36]. Such a co-packaging model is consistent with the observation that in small dsRNA viruses, such as partitiviruses, the number of RdRP molecules packaged during assembly is similar to the number of packaged genome segments [68,69]. Interestingly, recent studies on cypoviruses, which are members of the family *Reoviridae*, show that only ten instead of twelve polymerase complexes are visible in each particle [7,10], indicating that protein-RNA interactions also play an important role in genome packaging in these multi-layered dsRNA viruses.

**Fig 8:**
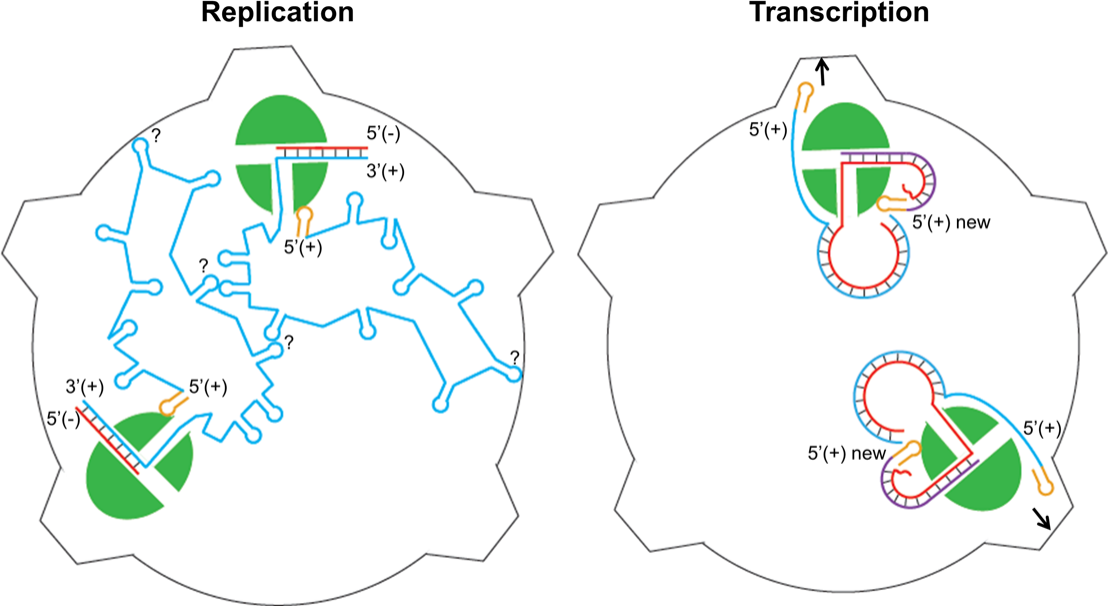
Proposed model for hPBV RdRP replication (left) and transcription (right) inside the viral capsid. The hPBV RdRP molecules are displayed in green. The blue and red lines correspond to the (+) and (-)strand respectively with the nascent (+)strand RNA represented in purple (right). The 5’-terminal stem loop structure is displayed in yellow. How exactly the two ssRNA molecules interact with each other and also with the viral CP during assembly and replication is not yet clear, as indicated by the questions marks in the figure on the left. The parental (+)strand RNA is separated from the template RNA during transcription and directed towards a pore in the viral capsid (right).

A secondary structure prediction of both segments of the hPBV genome by the program ViennaRNA Package 2.0 [55] predicted that both PBV (+)strand RNA segments begin with an AU-rich (~80%), RNA stem-loop structure (S7 Fig). The low melting temperature associated with the AU-rich 5’-(+)UTR should facilitate the denaturation of the dsRNA genome into an ssRNA template during the initiation of the transcription. We speculate that the specific binding of the hPBV polymerase to the 5’-(+)UTR may play two critical roles during the viral life cycle. First, the 5’-(+)UTR may function as a recognition element to ensure the specific packaging of the viral RNAs during virus assembly. Second, in the mature virion, this binding could help to direct the 3'-end of the genomic (-)strand RNA into the template entry channel of the RdRP to initiate the transcription (Fig 8). It has been reported that polymerases from members of the *Reoviridae* family utilize a 5’-cap binding activity to gain access to the 3’-end of the (-)RNA template during transcription initiation [21]. PBV does not encode a capping enzyme, and its genomic (+)strand RNA is not expected to have a cap. Nevertheless, the 5’-(+)UTR sequence of the PBV genome could play a similar role as the reovirus mRNA cap to facilitate transcription initiation and also to ensure that only the (-)strand RNA template is selectively used as a template for viral mRNA production.

It is also worthwhile to point out that our recombinant hPBV capsid does not undergo proteolytic cleavage as was previously reported for the rabbit PBV capsid, in which the first 65 residues of the capsid protein were removed from the assembled particles presumably due to self-cleavage [34]. Expression of the hPBV CP without the structurally flexible N-terminal peptide (corresponding to the first 65 residues in the rabbit PBV CP) produced recombinant capsids that were indistinguishable from the WT capsid (Fig 6A). As the cleavage site residues are conserved in the human PBV CPs, it is unclear whether the lack of proteolytic cleavage is due to strain difference or perhaps the use of a different expression host (*i*.*e*. *E*. *coli* vs. insect cells).

Our findings lead us to propose a model for PBV assembly and genome replication in which the RdRP first binds to one of the PBV positive-sense RNAs utilizing the AU-rich sequence located at the 5'-(+)UTR (Fig 8). The same genome segment is also acted upon by the PBV CP that is in the process of forming a virus capsid, most likely utilizing the highly flexible N-terminal region of the protein [34]. The capsid then assembles around the two genome segments which are bound to two separate RdRP molecules. Conversion of ssRNA to dsRNA occurs either in partially or fully assembled capsids resulting in transcriptionally active particles. Within the capsid the 5’-(+)UTR binding to the RdRP facilitates template strand selection and recognition during repeated transcription events. Transcription of the PBV genome segments proceeds in a semi-conservative manner where the parental (+)strand is replaced by the newly synthesized strand (Fig 5A, right). To fill in the gaps in our model, further experiments are needed to elucidate how the RdRP recognizes the 5’-(+)UTR and to map the molecular signals responsible for the specific packaging of the PBV genome by the viral CP. We expect that future studies of the PBV will help to define a new paradigm for the assembly and replication of small dsRNA viruses in which the viral RdRP most likely functions independently of the viral capsid.

## Materials and Methods

### Cloning, Protein Expression, and Purification

The gene encoding the RdRP of the hPBV Hy005102 strain (534 aa, ~62 kDa with either N-terminal or C-terminal 6xHis tag and without His-tag) was cloned into a pET28b(+)-vector (Novagen). The corresponding UTRs were not included in the cloned sequence. The resulting constructs were transformed into a Rosetta 2 strain of *E*. *coli* (Novagen) and expressed by isopropyl β-D-thiogalactopyranoside (IPTG) induction. The cells where then pelleted before being resuspended and sonicated in a lysis buffer composed of 50 mM Tris-HCl pH 7.5, 500 mM NaCl, 10% glycerol, 5 mM imidazole, 5 mM β-mercaptoethanol, 20 μg/ml phenylmethylsulfonyl fluoride (PMSF) or 1 mM Pefablock, 50 μg/ml ribonuclease A, and 10 μg/ml DNase 1. When the RdRP was purified for activity assays glycerol, β-mercaptoethanol and ribonuclease A were omitted and lysozyme was included. The lysate was clarified by centrifugation at 20,000 ×*g* for 60 minutes. The hPBV histidine tagged RdRPs were purified using a nickel-NTA (Thermo Fischer Scientific), HiTrap Heparin HP, and a Superdex 200 size exclusion column (GE Healthcare Life Sciences). The RdRP was eluted from the Superdex column in a buffer composed of 50 mM Tris-HCl pH 7.5, 300 mM NaCl, 10% v/v glycerol, 1 mM EDTA, 1 mM NaN_3_, and 5 mM β-mercaptoethanol. The purified RdRP was concentrated to 10 mg/ml for crystallization. When the RdRP was purified for the activity assays the Superdex column was omitted and the polymerase was stored in a buffer composed of 50% (v/v) glycerol, 50 mM Tris-HCl pH 8.0, 0.1 mM EDTA, 0.1% Triton X-100, ~150 mM NaCl at -20°C. The ΔLOOP RdRP was expressed and purified using the same protocol as the WT protein. For the untagged RdRP used for activity assays, a similar protocol was adopted except that the Ni-NTA-column was not used and HiTrap Q HP column purification step was added before the Heparin HP column purification (GE Healthcare).

For structure determination, the selenomethionine (SeMet) labeled RdRP was obtained by expressing the protein in M9 minimal media containing SeMet and a mixture of six other amino acids to prevent methionine synthesis [70]. The SeMet labeled protein was expressed and purified using the same protocol as the native protein.

### Crystallization and Data Collection

Crystals of the N-terminally histidine tagged hPBV RdRP were obtained by the hanging drop vapor diffusion method. The crystallization drop contained 1.5 μl of the RdRP solution and 0.5 μl of the mother liquor solution composed of 200 mM sodium acetate, 100 mM sodium cacodylate pH 6.5, and 30% (w/v) polyethylene glycol (PEG) 8000. Crystals appeared after incubation at 20°C for about 3 days and grew to full size (80 x 200 x 70 μm^3^) in approximately a week. The crystals were then transferred into a cryoprotectant solution (30% (v/v) glycerol, 200 mM sodium acetate, 100 mM sodium cacodylate pH 6.5, 30% (w/v) PEG 8000) and flash frozen in liquid nitrogen. The crystals were sent for data collection at the Beamline 4.2.2 at the Advanced Light Source, Lawrence Berkeley National Laboratory. The data were reduced and scaled using HKL2000 (Table 1) [71].

### Structure Determination

The heavy atom sites and experimental phases were determined by the AutoSol Wizard in the PHENIX software suite [72]. The protein model was built using PHENIX Autobuild, manually adjusted using COOT [73], and refined with phenix.refine. All of the structures presented were prepared using the program PYMOL unless otherwise specified (The PyMOL Molecular Graphics System, Version 1.2r3pre, Schrödinger, LLC). The coordinates have been deposited at the Protein Data Bank (PDB ID 5I61 and 5I62 for the full-length and ΔLoop RdRP, respectively).

### Production of RNA Molecules

The plasmids used for ssRNA and dsRNA production are presented in S2 Table. The full-length hPBV genome segment 2 (hPBV2) in a pMA-RQ-plasmid was synthesized by Life Technologies. The ϕ6 and hPBV specific ssRNAs were prepared by *in vitro* transcription using the T7 RNA polymerase and complementary DNA templates amplified by polymerase chain reaction (PCR). The primers used for hPBV2-specific cDNA production were hPBV2_T7_Forward (5’ CGCGTAATACGACTCACTATAGTAAAATTTTCGAATTTTATAATAATTAAG) and hPBV2_Reverse (5’ GCAGTTGGGACTGTTAGTCCCAATG) as well as hPBV2_Forward (5’ GTAAAATTTTCGAATTTTATAATAATTAAG) and hPBV2_T7_Reverse (5’ CGCGTAATACGACTCACTATAGCAGTTGGGACTGTTAGTCCCAATG) for (+)strand and (-)strand ssRNA production, respectively. For ϕ6 specific cDNA production primers T7-1 and 3’end [74] were used. For the production of the truncated PBV (+)strand ssRNAs the hPBV2_T7_Forward primer was replaced with PBV2_T7_5'_34 (5’ CGCGTAATACGACTCACTATAGGAGTTTAATAGTTTATCACAACTTAAAAGTG) or PBV2_T7_5'_646 (5’ CGCGTAATACGACTCACTATAGGGTGGCGAGGCCAGGAG) for Δ1–33 and Δ1–645, respectively. The produced ssRNAs were purified using chloroform extraction and successive precipitations with 4 M LiCl and 0.3 M sodium acetate, pH 6.5. The ϕ6 and hPBV specific ssRNAs were converted to dsRNA using the ϕ6 RdRP as described in [74]. The reaction mixtures were incubated for 1–3 hours at 30°C and the dsRNA was purified from the ssRNA by stepwise precipitation with 2 M and 4 M LiCl as described previously [75]. The ϕ6 genomic dsRNA was purified using Trizol/chloroform (5:1) extraction followed by successive precipitations with 44% (v/v) isopropanol, 4 M LiCl, and 0.3 M sodium acetate pH 6.3. The purified RNA was washed with cold 70% (v/v) ethanol and dissolved to sterile Milli-Q water.

### RdRP Activity Assays

The replication, transcription and TNTase activities of the hPBV RdRP were assayed in 6% (w/v) PEG 4000, 20 mM NH_4_Ac, 0.1 mM EDTA, 2 mM MgCl_2_, 0.1% (v/v) Triton X-100, 50 mM HEPES-KOH pH 7.5 and 0.4 U/μl RNase inhibitor RiboLock (Thermo Scientific), typically in 10 μl reaction volumes, using 55 nM concentrations of the WT or ΔLOOP polymerase and equimolar amounts of the RNA strands. The conditions were not stringent since PBV RdRP exhibited replication and transcription activity also in different divalent cation conditions (MgCl_2_ and MnCl_2_) and NTP concentrations. When back-priming and transcription time course reactions were assayed the RdRP amount was increased eight fold (without changing the molarity of the template RNA). A final concentration of 0.2 mM NTPs was used in the replication and transcription reactions. The TNTase activity was assayed in the presence of 0.03 μM UTP. For the identification of newly synthetized RNAs the reactions were supplemented with (α-^33^P) labeled UTP (0.1 μCi/μl reaction, Perkin-Elmer, 3000 Ci/mmol) or (γ-^32^P) labeled GTP (0.2–0.3 μCi/μl reaction, Perkin-Elmer, 6000 Ci/mmol). The reactions were incubated for 1 hour at 37°C and stopped with the addition of 2×U loading buffer (8 M Urea, 10 mM EDTA, 0.2% (w/v) SDS, 6% (v/v) glycerol, 0.05% (w/v) bromophenol blue, 0.05% (w/v) xylene cyanol). For the evaluation of the back-priming activity, the reaction products were boiled for 3 minutes to denature the sample. The reaction mixtures were analyzed by gel electrophoresis in 0.8 or 1.2% (w/v) agarose. The gels were dried and the signals were collected on imaging plates (Fujifilm), which were subsequently scanned using Typhoon TRIO Imager (GE Healthcare). Quantification was done using AIDA Image Analyzer.

### Co-expression Experiments

The gene encoding the CP of the hPBV Hy005102 strain (552 aa, 62 kDa) was cloned into a pET19b(+) vector without the corresponding UTRs. This construct was co-transformed along with the hPBV RdRP gene in a pET28b(+) vector into a Rosetta 2 strain of *E*. *coli*. The expression of both proteins (*i*.*e*. untagged CP and His-tagged RdRP) was then induced by the addition of IPTG. The cells were pelleted, resuspended, sonicated, and clarified in as described for the WT RdRP. After clarification, the supernatant was run through a nickel-NTA column to remove any free RdRP. The VLPs were subsequently purified using density gradient ultracentrifugation in a CsCl gradient of 1.1–1.4 g/cm^3^. The ultracentrifugation was performed using a SW41 Ti rotor (Beckman Coulter) at 35,000 rpm for 6 hours, after which the light-scattering VLP-zone was collected. Western blots were carried out using an anti-hPBV CP antibody obtained from Pacific Immunology (Ramona, CA, US) and an anti-6xHis antibody obtained from ThermoFisher (Houston, TX, US). VLP formation was confirmed using TEM (JEOL 2010, Japan) as previously described [76].

### Gel Shift Assays

The RNA oligos for the gel shift assays were commercially purchased from Sigma-Aldrich. The three RNA oligonucleotides used for these experiments were the PBV 5’-(+)UTR (5’-GUAAAAUUUUCGAAUUUUAU-3’), PBV 3’-(+)UTR (5’-GGACUAACAGUCCCAACUGC-3’), and a nonsensical CA repeat (5’-CACACACACACACACACACA-3’). The two PBV2+ ssRNA molecules, including the full-length PBV2+ and a deleted version PBV2+(Δ1–645), were made by *in vitro* transcription as described above. RNA oligos and PBV2+ ssRNA molecules were labeled with γ-^32^P-ATP using T4 polynucleotide kinase (New England Biolabs) at 37°C for 30 minutes. The labeling was halted by the addition of 5 μl of 500 mM EDTA. The labeled oligonucleotides were then purified and desalted using a Sephadex G-50 Nick column (GE Life Sciences). The gel shift assays were conducted in 50 mM Tris-HCl pH 7.5, 50 mM KCl, 1 mM NaN_3_, 5 mM β-mercaptoethanol, and 10% (v/v) glycerol. A 1 nM concentration of ^32^P-labeled RNA was incubated with increasing concentrations of either the WT or the ΔLOOP hPBV RdRP for 30 min at room temperature. The samples were then loaded onto a 15% (w/v) native polyacrylamide gel and run at 50 V for 4 hours. The polyacrylamide gel setup was placed in an ice bath during the experiments to minimize heating. The radioactive RNA was visualized by phosphorimaging using the FujiFilm FLA-5000 imager and quantified using the program ImageGuage v4.0. The fraction of the RNA bound was then calculated as the amount of the bound RNA divided by the sum of the total RNA and plotted versus the corresponding concentration of the protein.

## Supporting Information

S1 Fig Gel filtration chromatograms of the WT and ΔLOOP hPBV RdRPs. The elution profiles of the WT and ΔLOOP RdRPs are displayed in red and blue, respectively. The elution positions of three reference proteins and their relative molecular weights are indicated above.
(TIF)

S2 Fig Multiple sequence alignment for mammalian and avian PBV polymerases. (A) Alignment of ten polymerase sequences from PBV infecting various hosts. As indicated by the color bar, conserved regions are shown in pink while poorly conserved regions are shown in purple. The seven polymerase motifs are highlighted in black boxes. The six highly variable regions are shown in blue boxes. Secondary structure assignments based on the hPBV polymerase are included and the coloring schedule is the same as in Fig 1. (B) The six highly variable regions mapped onto the hPBV polymerase structure. The variable regions are numbered from the N-terminus and their corresponding colors are indicated at the bottom.
(TIF)

S3 Fig RNA synthesis activities by hPBV RdRP proteins with and without fusion tags. Starting from the left are hPBV RdRP proteins with an N-terminal his-tag, a C-terminal his-tag, and without any fusion tag. The RNA synthesis activities were assayed in the presence of (α-^33^P) labeled UTP using three RNA templates: PBV2+ and PBV2- ssRNAs as well as PBV2 dsRNA. The main radiolabeled products detected were PBV2 dsRNA as shown on the left.
(TIF)

S4 Fig RNA transcription time course by the hPBV RdRP. Reaction products were collected from 0 minute to 240 minutes after reaction started. Templates used were the ϕ6 genomic RNA including RNA segments of three distinct sizes (L, M, and S). Transcription products made by ϕ6 RdRP were included in the first lane for comparison. The expected positions of dsRNA (L, M, S) and ssRNA (l+, m+, s+) products are indicated on the left of the gel.
(TIF)

S5 Fig The effect of terminal sequence on the transcription activity of the WT hPBV RdRP. The RNA templates applied are indicated at the bottom and the mobility of the RNA products on the left. The amount of the *in vitro* produced ϕ6-specific dsRNA (lanes 2 to 5) was approximately one third of the amount of the ϕ6 genomic dsRNA (lane 1). The 3'-terminal sequences of the transcription (-)strand templates are shown at the bottom. Additional terminal sequences of ϕ6 genomic RNA can be found in S1 Table.
(TIF)

S6 Fig Relative template usage of the hPBV RdRP. (a) RNA replication using ssRNA templates. RdRP activity was tested using PBV2+, ϕ6 s+, and a mixture of PBV2+ with ϕ6 s+. Equimolar amounts of each ssRNA and RdRP were applied in the competition reaction (3^rd^ lane). (b) RNA transcription using dsRNA templates. RdRP activity was tested using PBV2, ϕ6 S, ϕ6 L, and two mixtures of PBV2 with either ϕ6 S or ϕ6 L genomic segment. In the competition reactions (4^th^ and 5^th^ lanes) the amount of each RNA template was one-fourth of the equimolar amount of the RdRP. The mobilities of the expected dsRNA reaction products are indicated on the left.
(TIF)

S7 Fig Predicted secondary structure of the hPBV genomic (+)strand sequences. Left, PBV1+. Right, PBV2+. Both 5’ and 3’-ends of the RNA molecules are labeled. The predicted stem-loop structures at the 5’-end are highlighted in blue ovals.
(TIF)

S1 Table Terminal sequences of utilized viral genomic segments. (TIF)

S2 Table Viral RNA expression constructs. (TIF)

S1 References Supplemental references. (TIF)
