# Supplementary figures and images for: Initiation of RNA Polymerization and Polymerase Encapsidation by a Small dsRNA Virus
Source: PLoS Pathog. 2016 Apr 14;12(4):e1005523. doi: 10.1371/journal.ppat.1005523 (PMC4831847; doi:10.1371/journal.ppat.1005523)

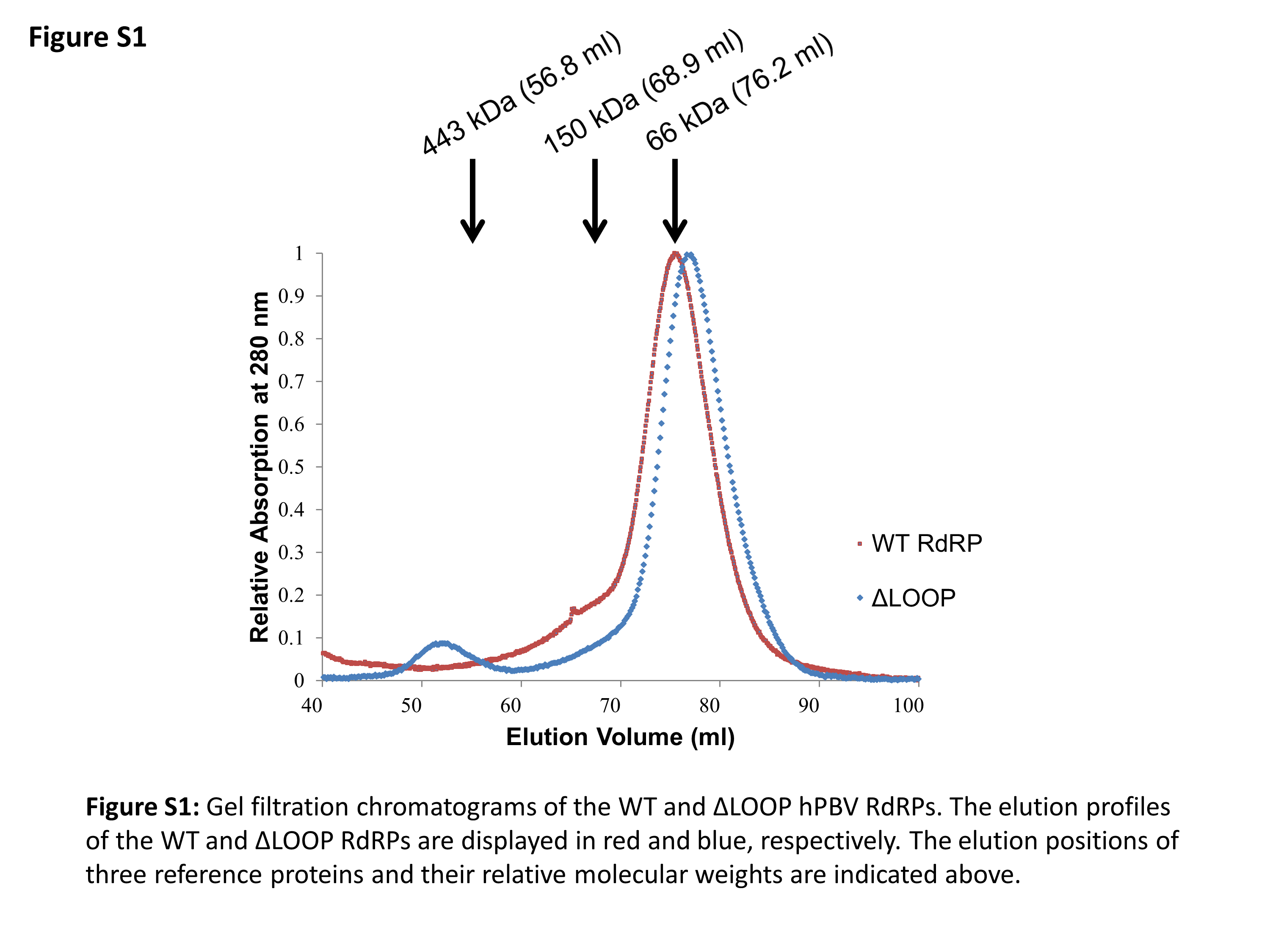

Supplement: S1 Fig — The elution profiles of the WT and ΔLOOP RdRPs are displayed in red and blue, respectively. The elution positions of three reference proteins and their relative molecular weights are indicated above. (TIF) [file ppat.1005523.s001.TIF]

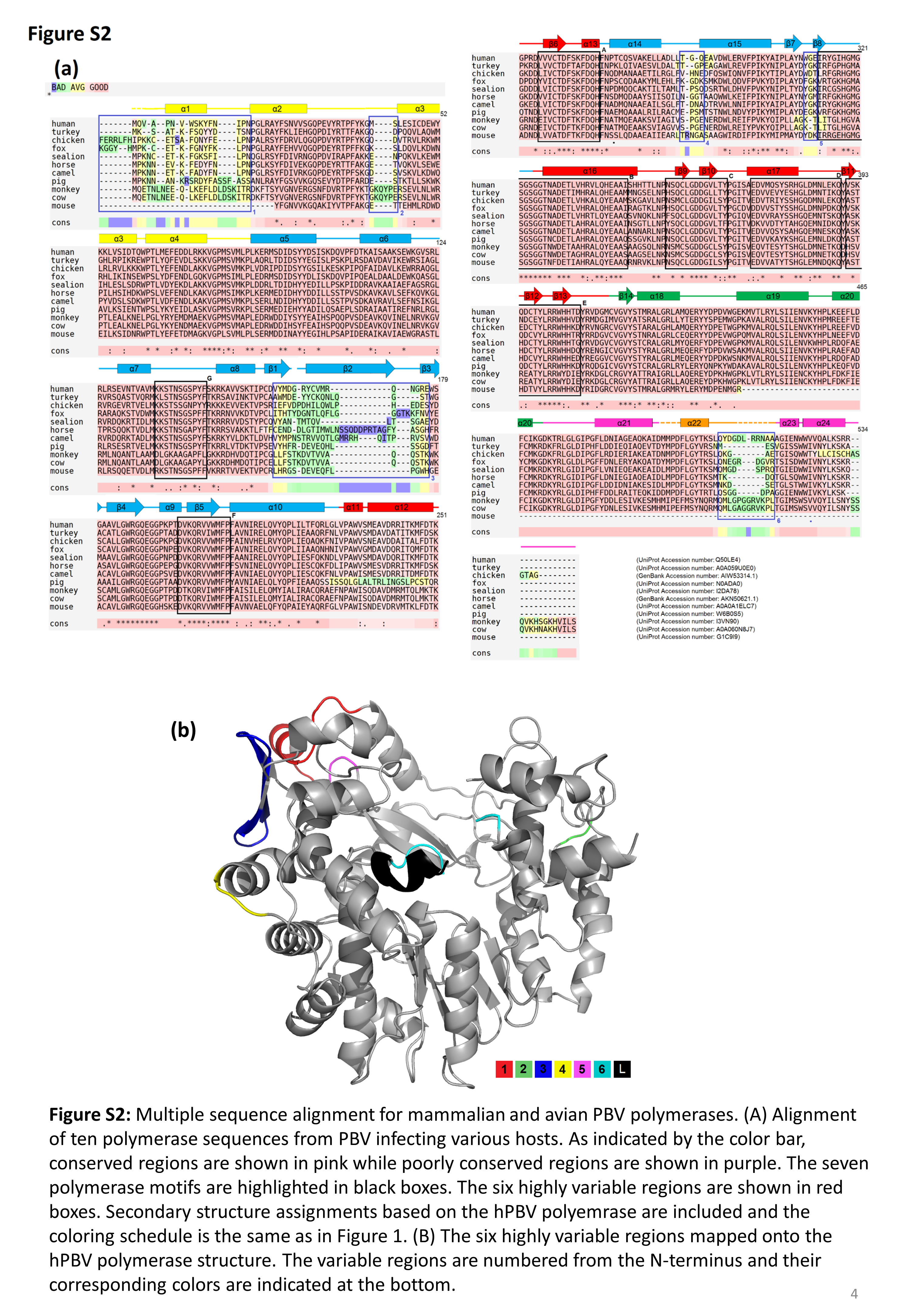

Supplement: S2 Fig — (A) Alignment of ten polymerase sequences from PBV infecting various hosts. As indicated by the color bar, conserved regions are shown in pink while poorly conserved regions are shown in purple. The seven polymerase motifs are highlighted in black boxes. The six highly variable regions are shown in blue boxes. Secondary structure assignments based on the hPBV polymerase are included and the coloring schedule is the same as in Fig 1. (B) The six highly variable regions mapped onto the hPBV polymerase structure. The variable regions are numbered from the N-terminus and their corresponding colors are indicated at the bottom. (TIF) [file ppat.1005523.s002.tif]

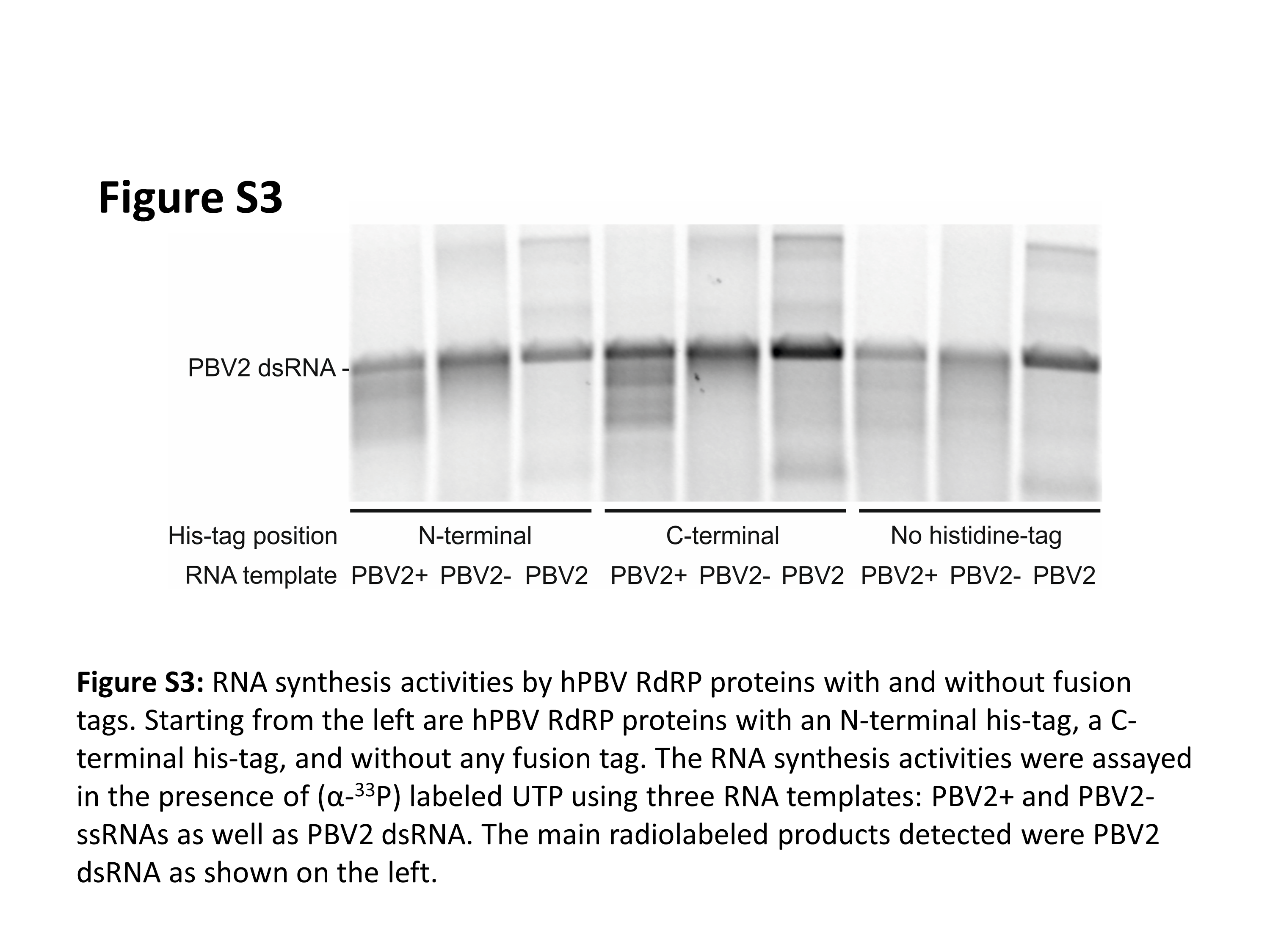

Supplement: S3 Fig — Starting from the left are hPBV RdRP proteins with an N-terminal his-tag, a C-terminal his-tag, and without any fusion tag. The RNA synthesis activities were assayed in the presence of (α-33P) labeled UTP using three RNA templates: PBV2+ and PBV2- ssRNAs as well as PBV2 dsRNA. The main radiolabeled products detected were PBV2 dsRNA as shown on the left. (TIF) [file ppat.1005523.s003.TIF]

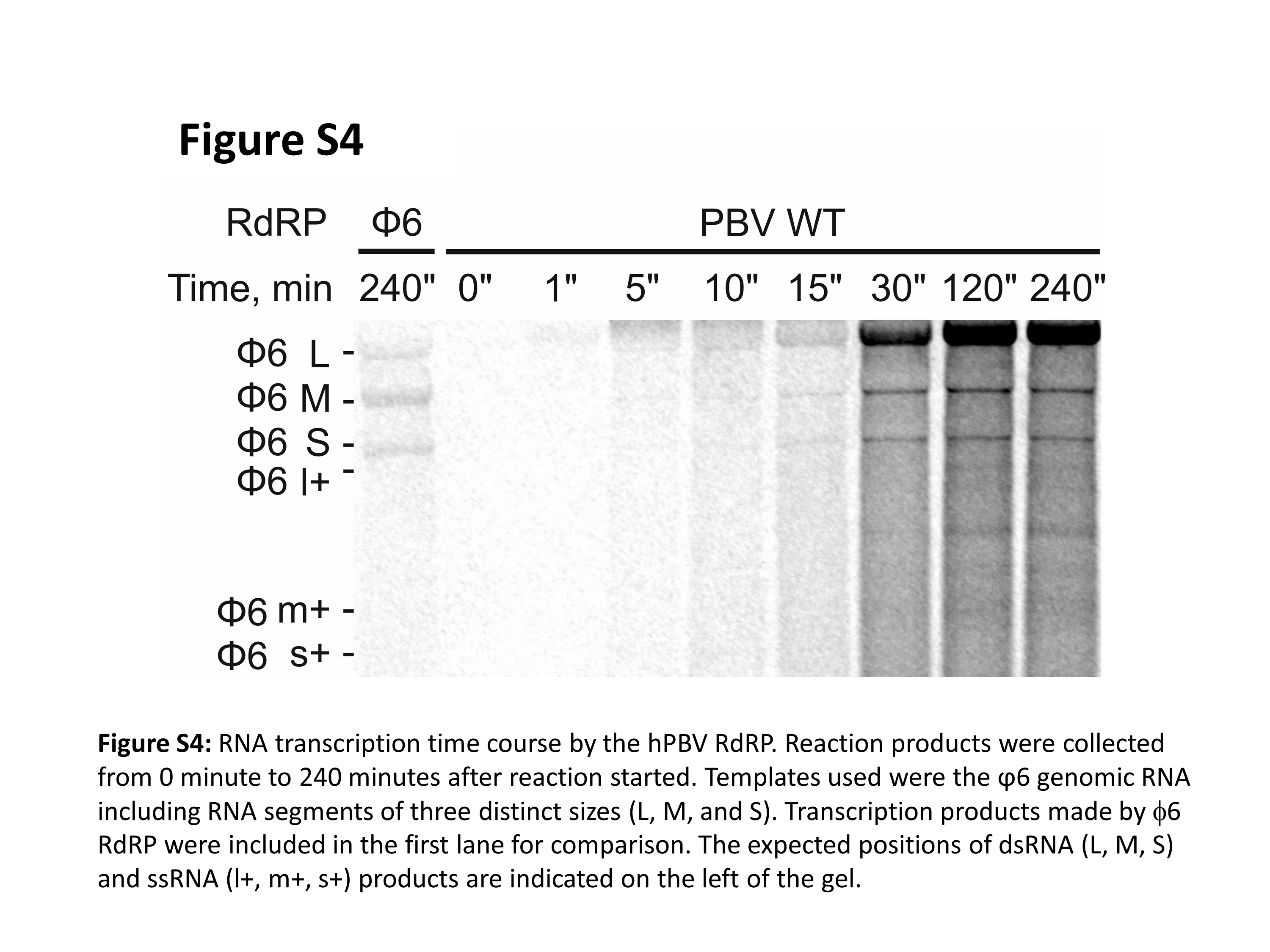

Supplement: S4 Fig — Reaction products were collected from 0 minute to 240 minutes after reaction started. Templates used were the ϕ6 genomic RNA including RNA segments of three distinct sizes (L, M, and S). Transcription products made by ϕ6 RdRP were included in the first lane for comparison. The expected positions of dsRNA (L, M, S) and ssRNA (l+, m+, s+) products are indicated on the left of the gel. (TIF) [file ppat.1005523.s004.TIF]

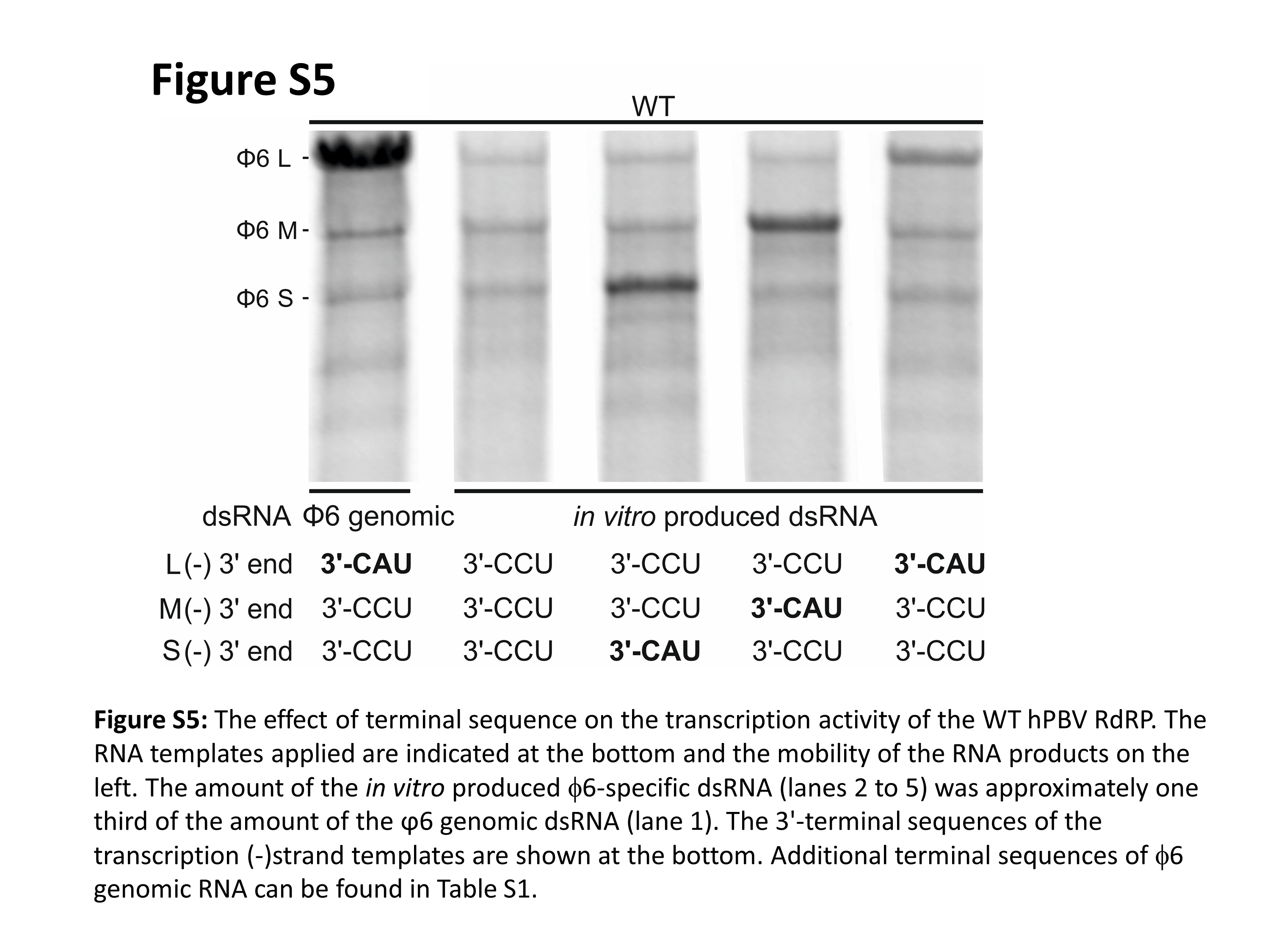

Supplement: S5 Fig — The RNA templates applied are indicated at the bottom and the mobility of the RNA products on the left. The amount of the in vitro produced ϕ6-specific dsRNA (lanes 2 to 5) was approximately one third of the amount of the ϕ6 genomic dsRNA (lane 1). The 3'-terminal sequences of the transcription (-)strand templates are shown at the bottom. Additional terminal sequences of ϕ6 genomic RNA can be found in S1 Table. (TIF) [file ppat.1005523.s005.TIF]

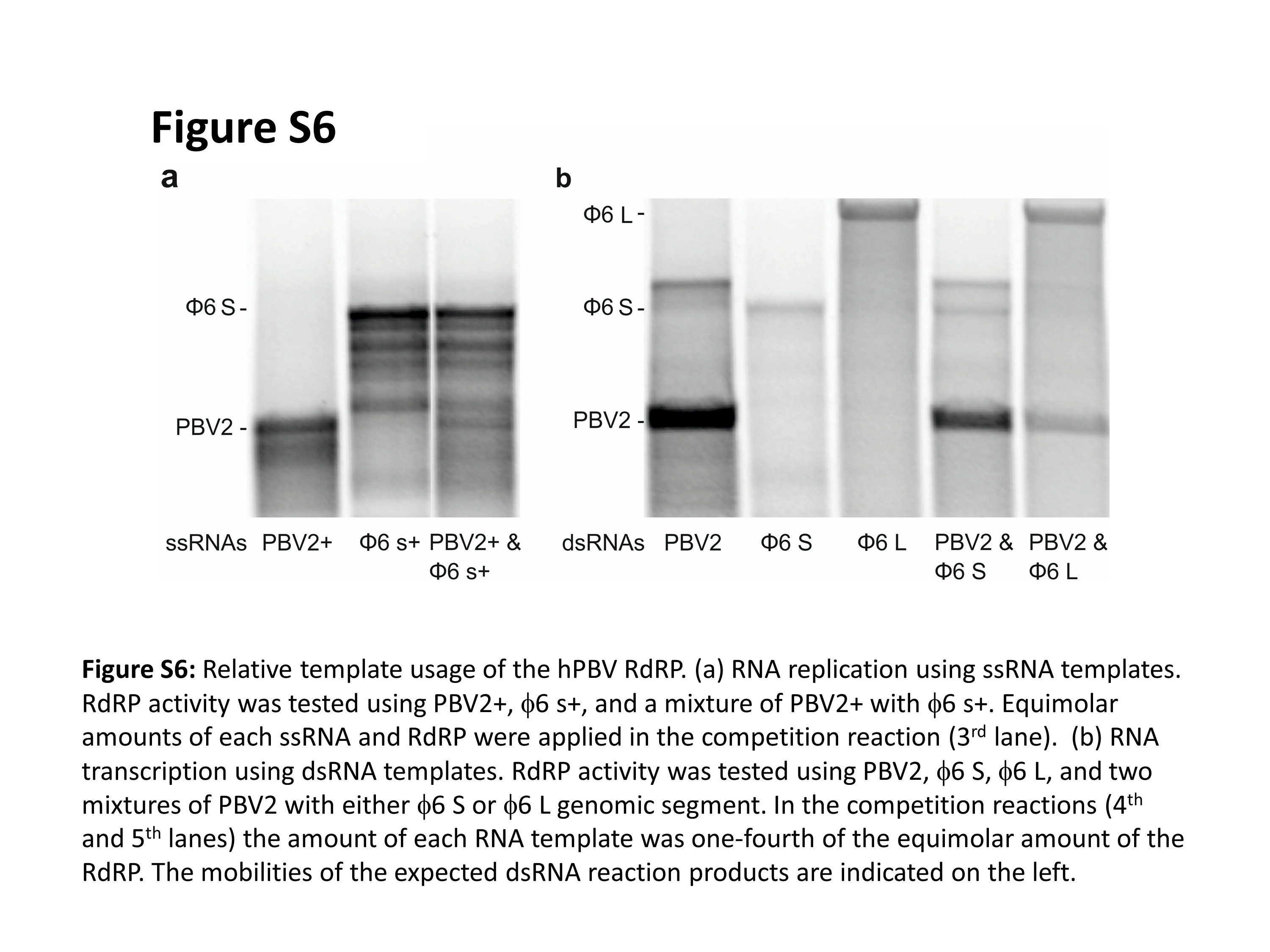

Supplement: S6 Fig — (a) RNA replication using ssRNA templates. RdRP activity was tested using PBV2+, ϕ6 s+, and a mixture of PBV2+ with ϕ6 s+. Equimolar amounts of each ssRNA and RdRP were applied in the competition reaction (3rd lane). (b) RNA transcription using dsRNA templates. RdRP activity was tested using PBV2, ϕ6 S, ϕ6 L, and two mixtures of PBV2 with either ϕ6 S or ϕ6 L genomic segment. In the competition reactions (4th and 5th lanes) the amount of each RNA template was one-fourth of the equimolar amount of the RdRP. The mobilities of the expected dsRNA reaction products are indicated on the left. (TIF) [file ppat.1005523.s006.TIF]

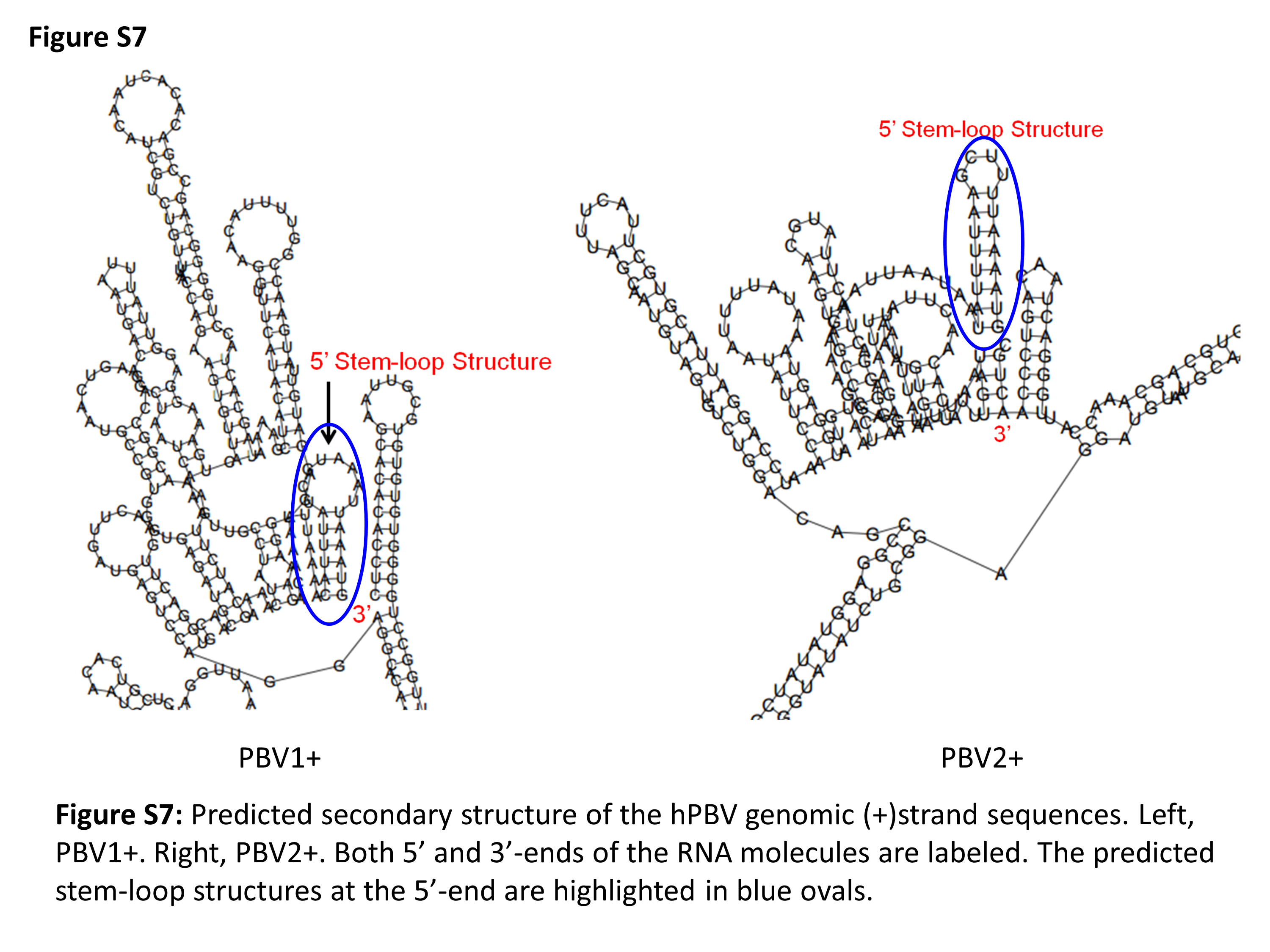

Supplement: S7 Fig — Left, PBV1+. Right, PBV2+. Both 5’ and 3’-ends of the RNA molecules are labeled. The predicted stem-loop structures at the 5’-end are highlighted in blue ovals. (TIF) [file ppat.1005523.s007.TIF]

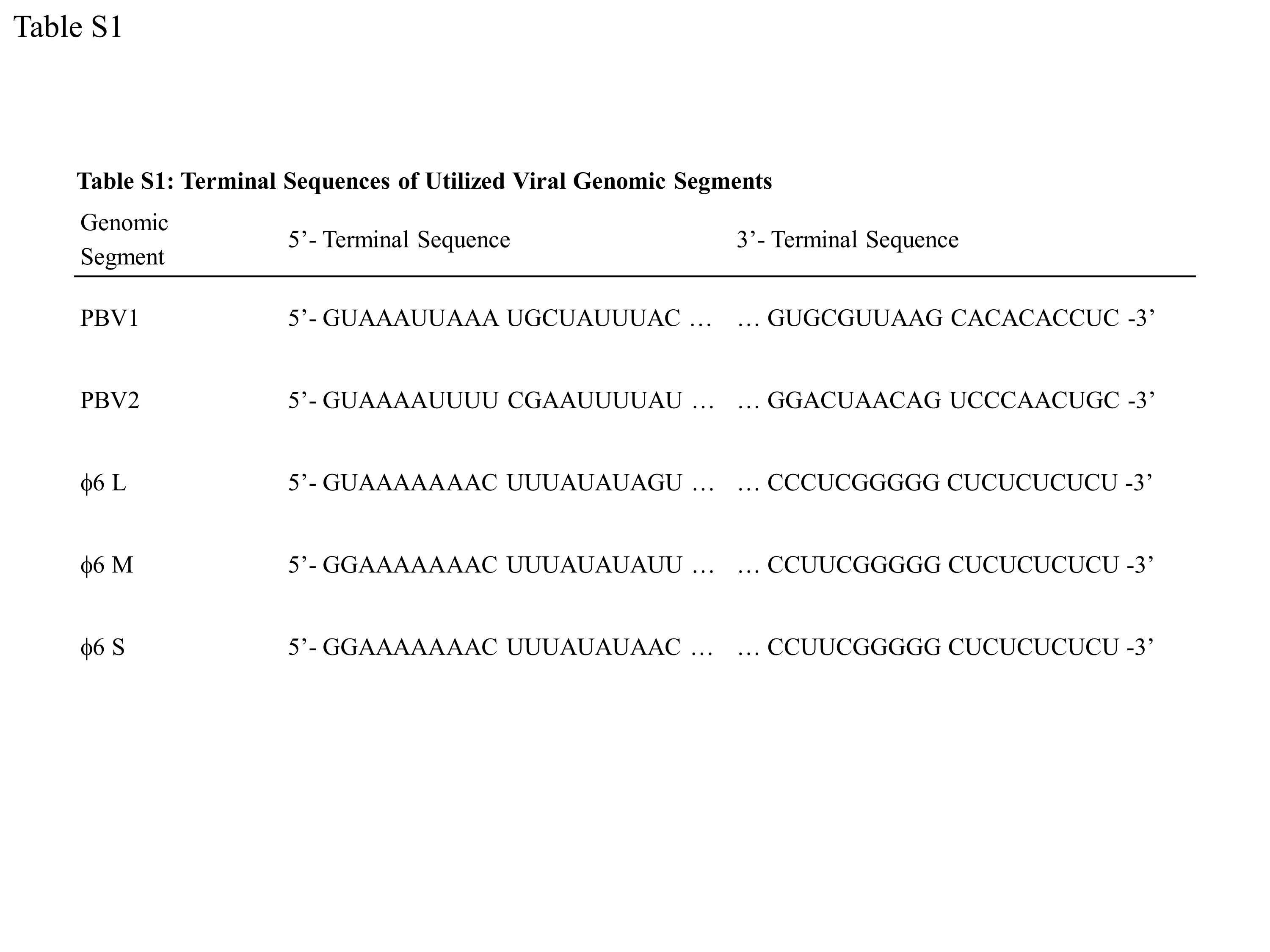

Supplement: S1 Table — (TIF) [file ppat.1005523.s008.TIF]

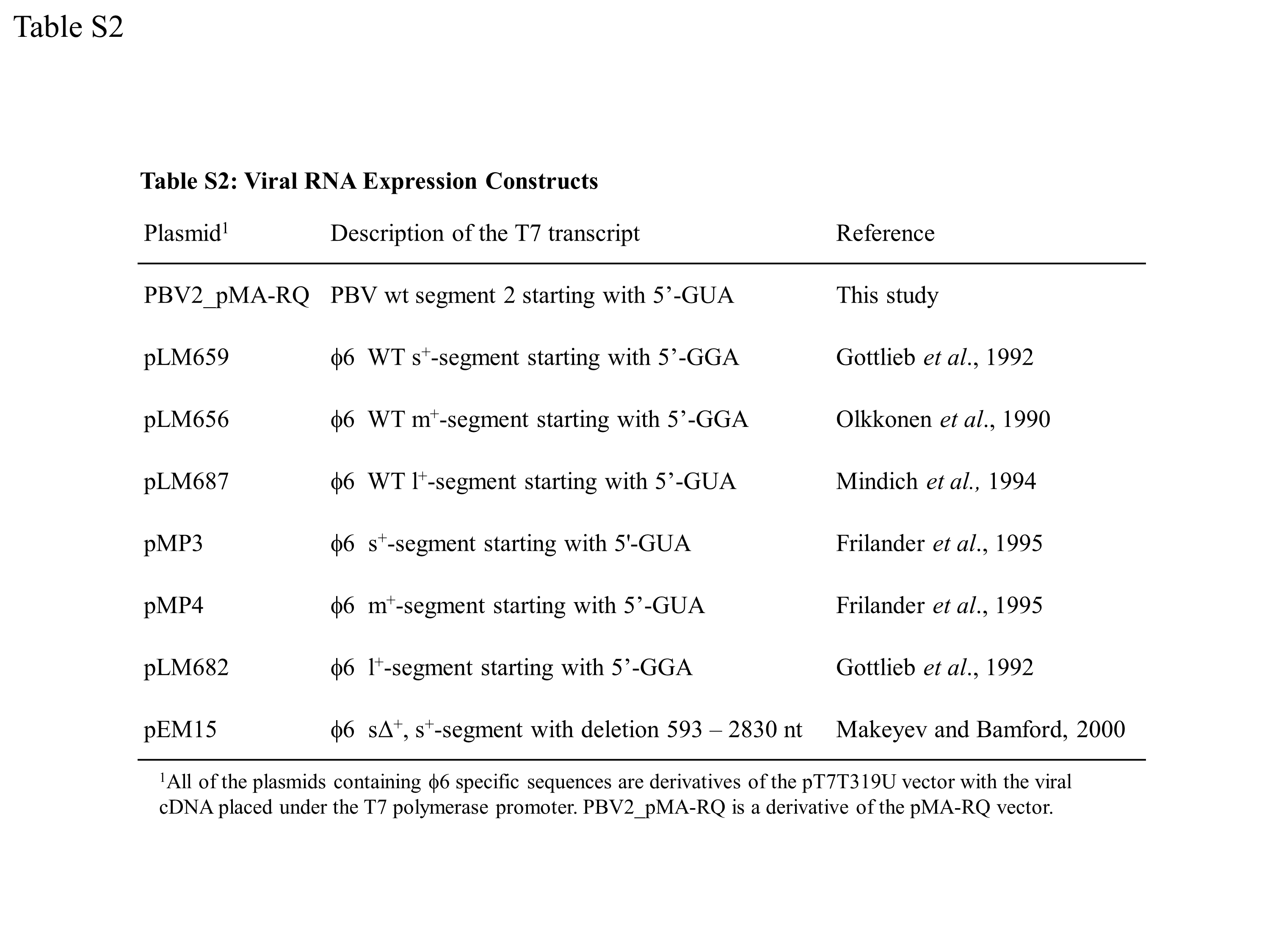

Supplement: S2 Table — (TIF) [file ppat.1005523.s009.TIF]

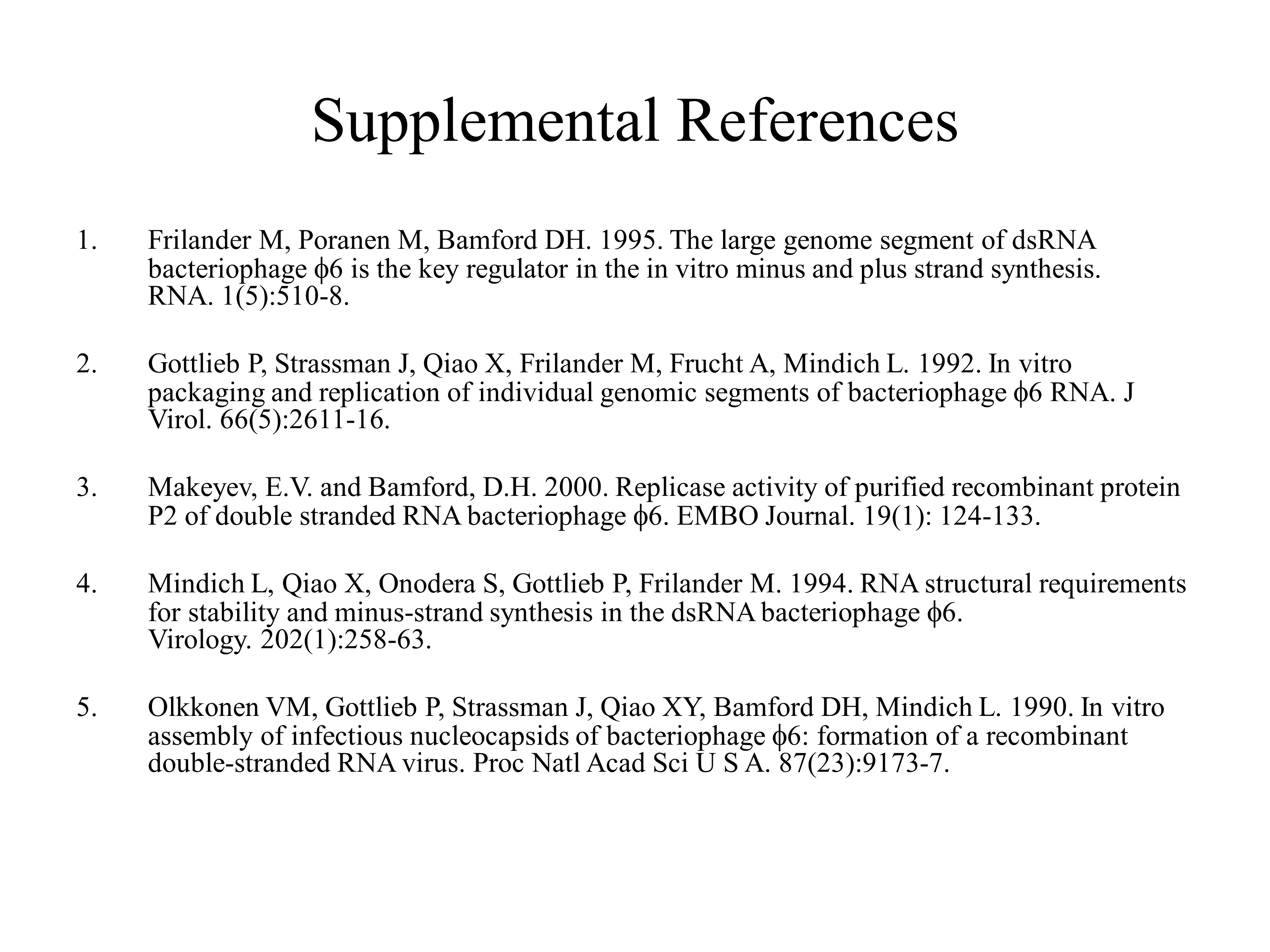

Supplement: S1 References — (TIF) [file ppat.1005523.s010.TIF]
